# Enhanced photosynthetic efficiency by nitrogen-doped carbon dots via plastoquinone-involved electron transfer in apple

**DOI:** 10.1093/hr/uhae016

**Published:** 2024-01-12

**Authors:** Xiuli Jing, Yankai Liu, Xuzhe Liu, Yi Zhang, Guanzhu Wang, Fei Yang, Yani Zhang, Dayong Chang, Zhen-Lu Zhang, Chun-Xiang You, Shuai Zhang, Xiao-Fei Wang

**Affiliations:** Apple Technology Innovation Center of Shandong Province, Shandong Collaborative Innovation Center of Fruit & Vegetable Quality and Efficient Production, National Key Laboratory of Wheat Improvement, College of Horticulture Science and Engineering, Shandong Agricultural University, Taian 271018, Shandong, China; Apple Technology Innovation Center of Shandong Province, Shandong Collaborative Innovation Center of Fruit & Vegetable Quality and Efficient Production, National Key Laboratory of Wheat Improvement, College of Horticulture Science and Engineering, Shandong Agricultural University, Taian 271018, Shandong, China; Key Laboratory of Agricultural Film Application of Ministry of Agriculture and Rural Affairs, College of Chemistry and Material Science, Shandong Agricultural University, Taian 271018, Shandong, China; College of Life Science, Shandong Agricultural University, Taian 271018, Shandong, China; Apple Technology Innovation Center of Shandong Province, Shandong Collaborative Innovation Center of Fruit & Vegetable Quality and Efficient Production, National Key Laboratory of Wheat Improvement, College of Horticulture Science and Engineering, Shandong Agricultural University, Taian 271018, Shandong, China; Apple Technology Innovation Center of Shandong Province, Shandong Collaborative Innovation Center of Fruit & Vegetable Quality and Efficient Production, National Key Laboratory of Wheat Improvement, College of Horticulture Science and Engineering, Shandong Agricultural University, Taian 271018, Shandong, China; Apple Technology Innovation Center of Shandong Province, Shandong Collaborative Innovation Center of Fruit & Vegetable Quality and Efficient Production, National Key Laboratory of Wheat Improvement, College of Horticulture Science and Engineering, Shandong Agricultural University, Taian 271018, Shandong, China; Yantai Goodly Biotechnology Co., Ltd, Yantai 264000, Shandong, China; Apple Technology Innovation Center of Shandong Province, Shandong Collaborative Innovation Center of Fruit & Vegetable Quality and Efficient Production, National Key Laboratory of Wheat Improvement, College of Horticulture Science and Engineering, Shandong Agricultural University, Taian 271018, Shandong, China; Apple Technology Innovation Center of Shandong Province, Shandong Collaborative Innovation Center of Fruit & Vegetable Quality and Efficient Production, National Key Laboratory of Wheat Improvement, College of Horticulture Science and Engineering, Shandong Agricultural University, Taian 271018, Shandong, China; Key Laboratory of Agricultural Film Application of Ministry of Agriculture and Rural Affairs, College of Chemistry and Material Science, Shandong Agricultural University, Taian 271018, Shandong, China; Apple Technology Innovation Center of Shandong Province, Shandong Collaborative Innovation Center of Fruit & Vegetable Quality and Efficient Production, National Key Laboratory of Wheat Improvement, College of Horticulture Science and Engineering, Shandong Agricultural University, Taian 271018, Shandong, China

## Abstract

Artificially enhancing photosynthesis is critical for improving crop yields and fruit qualities. Nanomaterials have demonstrated great potential to enhance photosynthetic efficiency; however, the mechanisms underlying their effects are poorly understood. This study revealed that the electron transfer pathway participated in nitrogen-doped carbon dots (N-CDs)-induced photosynthetic efficiency enhancement (24.29%), resulting in the improvements of apple fruit qualities (soluble sugar content: 11.43%) in the orchard. We also found that N-CDs alleviated *mterf5* mutant-modulated photosystem II (PSII) defects, but not *psa3* mutant-modulated photosystem I (PSI) defects, suggesting that the N-CDs-targeting sites were located between PSII and PSI. Measurements of chlorophyll fluorescence parameters suggested that plastoquinone (PQ), the mobile electron carrier in the photosynthesis electron transfer chain (PETC), was the photosynthesis component that N-CDs targeted. *In vitro* experiments demonstrated that plastoquinone-9 (PQ-9) could accept electrons from light-excited N-CDs to produce the reduced plastoquinone 9 (PQH_2_-9). These findings suggested that N-CDs, as electron donors, offer a PQ-9-involved complement of PETC to improve photosynthesis and thereby fruit quality. Our study uncovered a mechanism by which nanomaterials enhanced plant photosynthesis and provided some insights that will be useful in the design of efficient nanomaterials for agricultural/horticultural applications.

## Introduction

As the most important biological reaction on earth, photosynthesis directly or indirectly contributes to over 90% of the global agricultural yield [1]. It has been estimated that global agriculture production must double by 2050 to feed the continuously growing population [2]. To address this issue, enhancing photosynthetic efficiency would be of great value. Numerous artificial and semi-artificial strategies, such as cultivation up-grade [3, 4] and genetic engineering [5–8], have been proven to improve photosynthetic activities by directly or indirectly targeting photosynthetic components [2]. However, despite the demonstrated effectiveness of these strategies, photosynthetic theory indicates there is great potential for further improvement [8]. This indicates there are additional, entirely new, targets for crop yield improvement. To properly identify and harness these targets, both novel strategies and well-established technologies will be required.

In photosynthetic systems, several components can serve as direct targets for the enhancement of photosynthesis. Photosynthesis can be divided into three stages: light reaction, dark reaction, and sucrose synthesis [9]. Among them, the light reaction takes place in the thylakoid within the chloroplast organelle. There are several photosynthetic components, including photosystem II (PSII), photosystem I (PSI), plastoquinone (PQ), cytochrome *b_6_f* (Cyt *b_6_f*), ferredoxin (Fd), and plastocyanin (PC) [10]. Together these components constitute the photosynthetic electron transport chain (PETC) [11, 12]. In autotrophic plant, PQ-9 as an essential mobile electron carrier for photosynthesis diffuses and moves freely in the PQ pool until donating photoelectrons to the intermediate proton pump, completing the PETC [10, 13]. Furthermore, the oxidation of PQH_2_ in the quinol-oxidizing site of Cyt *b_6_f* complex is a rate-determining step in photosynthesis [14], which makes this photosynthetic component an excellent target for the enhancement of photosynthetic efficiency.

To directly target these components, nanomaterials are ideal candidates to reach the chloroplast organelle where photosynthesis occurs. In recent years, nanomaterials have emerged as important tools in agricultural biotechnologies due to their excellent electron donor/acceptor ability, good biocompatibility, good optical performance, and low cytotoxicity [15–19]. Recently, numerous nanomaterials have been used to improve photosynthetic efficiency and thereby crop yields [20, 21]. To explain this phenomenon, several hypotheses have been proposed: (i) Certain nanomaterials convert the UV-A/green light (not photosynthetically active) to blue/red light, which plants can employ [16]. (ii) Light-excited nanomaterials probably transfer their absorbed energy to the photosynthetic reaction center (RC) via Förster resonance energy transfer (FRET), resulting in improved light energy influx into RC [22]. (iii) Under light irradiation, nanomaterials produce photo-generated electron–hole pairs, thereby serving as electron donors and acceptors [23]; these photo-generated electrons are probably transferred to an unidentified photosynthetic component, thereby accelerating the electron flow in PETC [15, 21]. Recently, Giraldo *et al*. [15] found that fluorescent single-walled carbon nanotubes (SWNTs) could augment photosynthetic activity, possibly by accelerating electron transfer. It is worth noting that, in artificial/semi-artificial photosynthetic nanomaterial hybrids, nanomaterial-involved electron transport is a common mechanism [13, 24], indicating the possibility of electron transfer augmentation in plant photosynthetic systems. Collectively, considering the complexities of the photosynthesis system and the structural diversity of nanomaterials, any or all of the three proposed hypotheses could explain the nanomaterial-induced enhancements in photosynthesis. However, there is a lack of in-depth knowledge of the interplay among nanomaterials and photosynthesis, such as the receptors of energy/electrons from nanomaterials, which limits the design of efficient nanomaterials for photosynthesis enhancement.

Carbon dots (CDs) have emerged as highly promising carbon-based nanomaterials that exhibit various desirable characteristics, such as high biocompatibility, low toxicity, and low cost [25–27]. Numerous CDs applied by hydroponic culture, irrigation, or leaf-spraying were reported to benefit photosynthetic efficiency [20]. For example, hydroponic application with dual-emissive CDs on lettuce presented a maximum increase of 25% in electron transport rates [20]. Far-red CDs treatment on lettuce induced 51.14% and 24.60% increases in fresh and dry weights by enhancing photosynthetic activity [28]. Magnesium-nitrogen co-doped CDs at 300 mg·L^−1^ concentration increased the fresh biomass of rice seedlings by 70.60% through improving photosynthesis [29]. Interestingly, element-doping CDs usually exhibited excellent capacities in enhancing photosynthetic activity, probably due to element-doping-induced features in photoluminescence, charge delocalization and electron supply/transfer [25, 30–32]. Although several possible pathways for CD-induced photosynthetic activity enhancement were also proposed as discussed in nanomaterial-induced photosynthesis improvement, the corresponding mechanisms remained largely elusive. In this work, nano-sized nitrogen-doped CDs (N-CDs), synthesized by hydrothermal method with diethylenetriamine and citric acid as raw materials, were found to enhance photosynthetic efficiency by a mechanism in which PQ-9, the mobile electron carrier on the PETC, accept electrons from light-excited N-CDs to produce the reduced PQH_2_-9. This enhancement, enabled by an affordable and widely available material, will help improve plant growth and enhance fruit quality.

## Results

### Chemical and optical characterization of N-CDs

**Figure 1:**
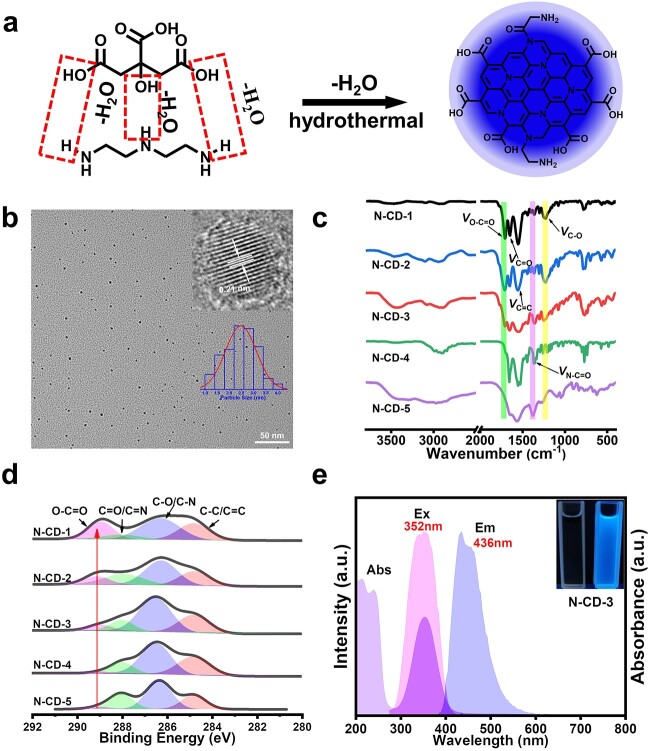
Chemical characterizations of N-CDs. (**a**) Illustration of the synthesis of N-CDs with diethylenetriamine and citric acid as raw materials. (**b**) HRTEM images and the size distribution of N-CD-3 (inserted image). (**c**) FT-IR spectra of N-CDs. (**d**) High-resolution XPS spectra of C_1s_ of N-CDs. (**e**) UV–Vis absorption (Abs), excitation (Ex), and emission (Em) spectra of N-CD-3.

First, motivated by the knowledge that nitrogen doping into CDs can induce charge delocalization and accelerate electron supply/transfer [31, 32], N-CDs (including N-CD-1, N-CD-2, N-CD-3, N-CD-4, and N-CD-5) were facilely prepared via one-pot hydrothermal treatments of diethylenetriamine and citric acid with incremental mole ratios (Fig. 1a). The particle sizes of N-CDs were first determined by transmission electron microscopy (TEM) and atomic force microscopy (AFM), as the size was one critical limitation for the movement of nanomaterials inside seedlings. The as-prepared N-CDs presented good mono-dispersity as microspherical particles with average diameters of 3.31 nm, 2.72 nm, 2.48 nm, 2.71 nm, 3.08 nm, respectively (Fig. 1b; Fig.S1, see online supplementary material). The AFM revealed the heights of N-CDs are 2.95 nm, 2.66 nm, 2.34 nm, 2.20 nm, 2.79 nm, respectively (Fig. S2, see online supplementary material). Based on the high-resolution TEM (HRTEM) images, these N-CDs displayed well-resolved lattice fringes with interplanar spacing ranging from 0.20 nm to 0.22 nm, respectively, which are consistent with the (100) diffraction facets of graphite (Fig. 1b; Fig.S1, see online supplementary material). These results proved the intrinsic nano-size features of N-CDs, which were smaller than the pore size (5 to 20 nm) of the cell wall [33], facilitating them to enter plant cells and function.

The chemical compositions of N-CDs were further characterized by Fourier transform infrared spectrophotometer (FT-IR) and high-resolution X-ray photoelectron spectrometer (XPS) tests, which clearly showed the peak changes with increasing diethylenetriamine dosage. As shown in FT-IR spectrum, the absorption peak at around 1710 cm^−1^ belonged to the stretching vibrations of carboxyl moiety (O=C-O), which decreased significantly from N-CD-1 to N-CD-5. The peaks at 1225 cm^−1^ can be attributed to C-O bond absorption. The peaks at 1380 cm^−1^ were assigned to the stretching vibration of C-N bond in amide groups. Interestingly, the weakening of the C-O bond absorption at 1225 cm^−1^ was accompanied by increasing C-N bond (in amide groups) peaks at 1380 cm^−1^ (Fig. 1c). More insights into the surface functional groups of N-CDs can be obtained from XPS analysis. The high-resolution C_1s_ spectra exhibited the existence of O-C=O, C=O/C=N, C-O/C-N, C-C/C=C. The peaks at 289.2 eV belonged to the O-C=O group (Fig. 1d) and the high-resolution N_1s_ spectra involved three peaks that are attributed to C-N (400.7 eV), graphitic N and pyridine/pyrrole N (Fig. S3c, see online supplementary material). As shown in Fig. 1d and Fig.S3 (see online supplementary material), the obvious increase in peak intensities of O-C=O group (289.2 eV) and decreases in the peak intensities of C-N amine groups (400.7 eV) were observed. Taken together, these results proved the successful N-doping inside CDs, and the changes suggested the decrease of carboxyl moieties and the increase of amine groups on the N-CDs surface from N-CD-1 to N-CD-5, which were agreed with the zeta potential changes (Fig. S4, see online supplementary material). Subsequently, the optical features were characterized. As shown in Fig. 1e and Fig.S5 (see online supplementary material), N-CDs displayed typical absorption at 220 nm, which belongs to π-π* transition of aromatic sp^2^ domains in cores and the strong absorption at about 350 nm was ascribed to the n-π* transition of the p-π orbit between the graphitic N/C–O and the conjugate structure from trapping excited-state energy [34]. Furthermore, these N-CDs exhibited obvious blue fluorescence (FL) emission (Fig. 1e; Fig.S5, see online supplementary material) under excitation, which was beneficial for observing their distribution and movement inside seedlings. Collectively, we successfully prepared N-doped carbon dots with nano-size, functional group-rich and promising optical features, and subsequently employed them to measure their roles in improving photosynthetic efficiency.

### N-CDs improve the photosynthetic efficiency

The uptake and translocation of CDs are critically involved in plant growth [35]. To investigate the absorption, transport, and distribution of the N-CDs in plants, the blue fluorescence characteristics of the N-CDs were used to visibly track their movement. After 2 d of hydroponic cultivation, N-CDs could be observed successively in the roots, stems, and lamina of seedlings incubated in the N-CDs enriched medium (Figs S6–8, see online supplementary material). Although the N-CDs presented no selective deposition on specialized organelles, a portion of the N-CDs were co-localized with chloroplasts (Figs S6 and S8, see online supplementary material), suggesting great potential in improving plant photosynthesis. Collectively, N-CDs were translocated throughout the plant body along a bottom-to-top (root-to-leaf) pathway.

**Figure 2:**
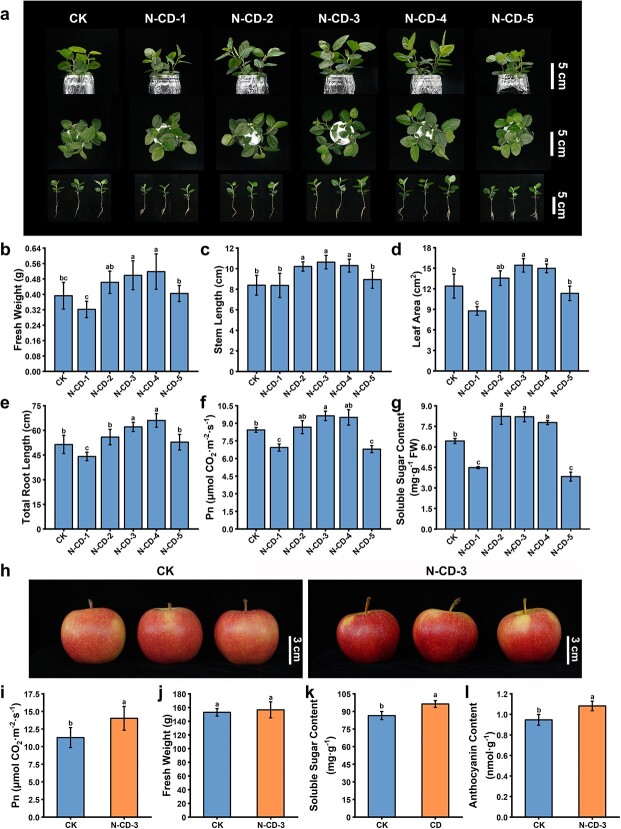
Effects of N-CDs in enhancing growth and photosynthesis of apple. (**a**) Effects of N-CDs (300 mg·L^−1^) on the *M. hupehensis* seedlings growth under light illumination (20.43 μmol·m^−2^·s^−1^) containing UV-A (λ_max_ 365 nm, 0.83 μmol·m^−2^·s^−1^). Pictures captured after 30 d of cultivation. (**b**–**g**) The fresh weight (b), stem length (c), leaf area (d), root length (e), Pn (f), and soluble sugar content (g) with/without the N-CDs treatment of seedlings. (**h**) Effects of N-CD-3 (1000 mg·L^−1^) on the fruit of *Gala/M9* under field conditions. (**i**) The leaf Pn values with/without treatment of N-CD-3. (**j**–**l**) The fresh weight (j), soluble sugar content (k), and anthocyanin content (l) with/without the N-CD-3 treatment of fruit. One-way analysis of variance (ANOVA) was used to analyse all data and error bars represent the SD from more than three biological replicates with three parallel experiments. Different lowercase letters represent significant differences (*P* < 0.05, Tukey test).

To investigate whether N-CDs were involved in the photosynthesis process, *Malus hupehensis* seedlings were cultivated in Hoagland nutrient solution containing 300 mg·L^−1^ N-CDs. Notably, the previous study found that N-CDs could improve plant growth more significantly under light conditions containing excitation light, so UV-A light was added because N-CDs are UV-A light-excited nanomaterials [36]. Interestingly, N-doping ratio-dependent effects were found in the seedling growth status and several growth-related parameters (Fig. 2a–g). N-CD-2, N-CD-3, and N-CD-4 had significant positive effects on seedling growth, while the N-CD-1 and N-CD-5 treatments adversely affected growth relative to the control (Fig. 2a). The N-CD-3 treated groups showed significant increases in fresh weight, stem length, leaf area, and root length compared to the control (Fig. 2b–e). Furthermore, there was a 14.23% increase in net photosynthetic rate (Pn) and 27.49% increase in soluble sugar content in the N-CD-3-treated groups (Fig. 2f–g). Similar growth phenotypes were also found in N-CD-2/3/4-treated *Arabidopsis thaliana* seedlings (Fig. S9, see online supplementary material).

Considering the ability of N-CDs to significantly increase the photosynthetic efficiency of *M. hupehensis* seedlings, foliar sprays of N-CD-3 (1000 mg·L^−1^) on 8-year-old *Gala/M9* trees were performed to verify its effect in fruit quality. As shown in Fig. 2i, N-CD-3 treatment resulted in a significant increase in leaf Pn value by 24.29% as compared to control. Subsequently, to further determine the effect of N-CD-3 on the fruit quality, the relevant indices were examined during fruit ripening. Inspiringly, the soluble sugar content (11.43%) was significantly improved (Fig. 2k). Notably, the N-CD-3-treated apple fruits had redder skin with a 14.36% increase in anthocyanin content compared to the control group (Fig. 2l; Fig.S10c–g, see online supplementary material). On the other hand, no significant changes were found in single fruit weight, fruit shape index, fruit luster, and hardness (Fig. 2h and j; Fig. S10a and b, h–l, see online supplementary material). Collectively, N-CD-3 enhanced photosynthesis efficiency and thereby plant growth and fruit quality.

### N-CDs-induced photosynthesis enhancement partially depends on electron transfer from N-CDs to chloroplasts

**Figure 3:**
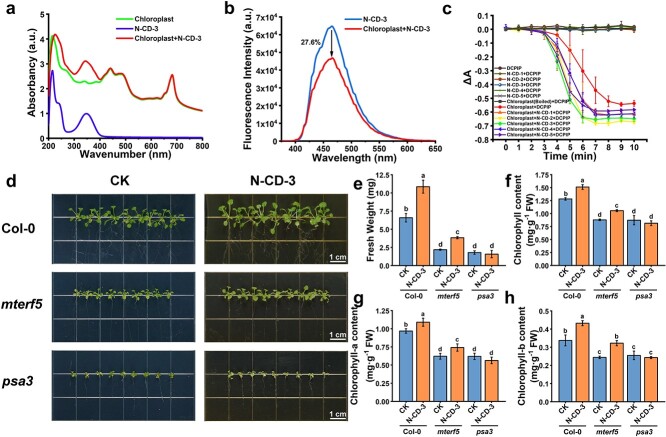
Occurrence of electron transfer progress between photosystem and N-CDs. (**a**) UV–Vis absorbance of chloroplasts, N-CD-3, and N-CD-3 + chloroplast. (**b**) FL spectra of N-CD-3 and N-CD-3 + chloroplast under 365 nm excitation. (**c**) Hill activity of DCPIP, N-CDs + DCPIP, chloroplast (boiled) + DCPIP, chloroplast + DCPIP and chloroplast + N-CDs + DCPIP under saturating light (150.62 μmol·m^−2^·s^−1^) containing UV-A (λ_max_ 365 nm, 4.06 μmol·m^−2^·s^−1^). One-way analysis of variance (ANOVA) was used to analyse all data and error bars represent the SD from more than three biological replicates with three parallel experiments. Different lowercase letters represent significant differences (*P* < 0.05, Tukey test). (**d**) Effects of N-CD-3 on the growth of Col-0, *mterf5* and *psa3* mutants under light illumination (20.43 μmol·m^−2^·s^−1^) containing UV-A (λ_max_ 365 nm, 0.83 μmol·m^−2^·s^−1^) in sucrose-free 1/2-strength Murashige and Skoog (1/2 MS) medium. (**e**–**h**) Effects of N-CD-3 on fresh weight (e), chlorophyll content (f), chlorophyll a content (g), and chlorophyll b content (h) of Col-0, *mterf5*, and *psa3* mutants. Pictures captured after 30 d of cultivation. One-way analysis of variance (ANOVA) was used to analyse all data and error bars represent the SD from three biological replicates with three parallel experiments. Different lowercase letters represent significant differences (*P* < 0.05, LSD test).

Because N-CDs can significantly increase the Pn values of apple seedlings and trees, follow-up experiments were conducted to investigate the mechanism of photosynthetic efficiency improvement. Notably, the measurements of stomatal apertures showed no apparent differences between the N-CD-3-treated group and the control (Fig. S11, see online supplementary material), indicating that stomatal parameters were not the main targets of N-CD-3 for photosynthesis enhancement. The photosynthetic efficiency enhancement may come from changes in the electron transfer process [15]. To test this, the electron transfer process was examined. Among the tested N-CDs, N-CD-3 had the best photosynthesis-enhancing effect (Fig. 2), so N-CD-3 and N-CD-3 + chloroplast composites were used in this investigation. Compared to the control, a significant increase in the absorption spectrum of the N-CD-3 + chloroplast composites in the range of 300 nm to 370 nm was observed (Fig. 3a). This was ascribed to the absorption of N-CD-3, suggesting that the energy harvested by N-CD-3 was consumed as fluorescence, heat dissipation, and/or energy/electron transfer, and the energy/electron transfer from N-CD-3 to chloroplasts probably augmented photosynthetic efficiency. Under 365 nm excitation, there was a 27.6% decrease in emission intensity of the N-CD-3/chloroplast composites compared to N-CD-3 alone (Fig. 3b), suggesting energy/electron was being transferred from N-CD-3 to the chloroplasts in the form of FRET or electron transfer. To prove the occurrence of the electron transfer process, saturating light (150.68 μmol·m^−2^·s^−1^) with an intensity higher than the light saturation point was used in the Hill reaction to measure the reduction rate of 2,6-dichlorophenolindophenol (DCPIP) and determine photosynthetic activity (Fig. S12, see online supplementary material) [29]. Under saturating light, chloroplasts would not be able to accept excess energy in the form of N-CDs-induced light conversion or FRET, but would accept electrons via PETC. As expected, the N-CD-3 + chloroplast composites exhibited significantly higher DCPIP reduction relative to chloroplasts alone, with maximum increases of 25.09% and 20.75% for N-CD-2 and N-CD-3 at 10 min, respectively (Fig. 3c). Meanwhile, N-CDs alone or inactivated chloroplasts (boiled) could not lead to DCPIP reduction (no significant changes in OD_600_ values). These results suggested that the electron transfer pathway from N-CDs to chloroplasts was partially, if not entirely, responsible for the photosynthetic efficiency enhancement. Having established the electron transfer link between N-CDs and chloroplasts, we subsequently investigated the capability of N-CDs to reach the thylakoid, where photosynthesis takes place in chloroplasts. The distribution of N-CD-3 in leaves was observed by bio-transmission electron microscopy (Bio-TEM) (Fig. S13, see online supplementary material). Numerous N-CD-3 (black particles) were found to be embedded in the thylakoid, suggesting the electron transfer process probably occurred in the thylakoid membrane.

### N-CD-3 alleviates *mterf5* mutant-modulated PSII defects, not *psa3* mutant-modulated PSI defects

Considering that N-CDs-induced photosynthesis enhancement may be partially dependent on electron transfer from N-CDs to the components in the chloroplast, the next step was to investigate the active electron acceptor in the N-CDs/chloroplasts interplay that underlies the N-CDs-induced photosynthetic improvement. There are several photosynthetic components involved in the photosynthetic electron transport chain, including the PSII, Cyt *b_6_f*, PSI, PQs, and ATP synthase, all of which could act as electron acceptors [10]. To verify the sites where N-CDs affected the photosynthesis system, the At4g14605/MTERF5 T-DNA insertion mutant of *A. thaliana* with a defect in the PSII function was studied [37]. The chloroplast protein mTERF5 is a transcriptional pausing factor that mediates transcriptional pausing and promotes the transcription of the chloroplast psbEFLJ manipulator, the absence of which leads to retarded plant growth, leaf yellowing, and a significant decrease in PSII activity. Under light illumination containing UV-A, better growth performances were observed in the N-CD-3-treated *mterf5* mutants compared to the untreated mutants no matter in sucrose-free and sucrose-containing medium (Fig. 3d; Fig.S14a, see online supplementary material). The results indicated significant enhancements in fresh weight and chlorophyll content (Fig. 3e–h; Fig.S14b–e, see online supplementary material), demonstrating that N-CD-3 could partially alleviate the photosynthesis defects of *mterf5* mutants. Taken together, by comparing the performances of the Col-0, *mterf5* mutants, and N-CD-3-treated mutants, N-CD-3 acts on the photosynthetic electron transport process and probably functions as a complement of PSII or the downstream of PSII. To provide further insight into the sites affected by N-CDs, *psa3* mutants that have a defect in PSI function were employed [38, 39]. PSA3 protein is a PSI assembly factor that functions in the assembly of PSI and the maintenance of PSI function, and its absence can cause impaired PSI assembly and decreased PSI activity. Similar growth performances were found between N-CD-3-treated *psa3* mutants and untreated mutants (Fig. 3d–h), indicating that N-CD-3 had little effect on the PSI. Therefore, N-CD-3 complements the PSII, but not the PSI on the PETC.

### PQ-9 accepts electrons from light-excited N-CD-3

**Figure 4:**
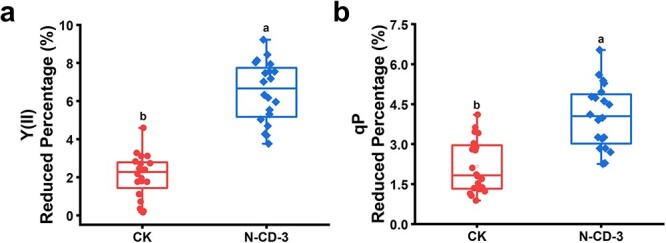
Electron transfer process from light-excited N-CD-3 to PQ-9 *in vivo*. (**a**) The reduced percentage of Y(II) values before and after the transient addition of UV-A light (λ_max_ 365 nm, 2.48 μmol·m^−2^·s^−1^) with/without treatment of N-CD-3 in *M. hupehensis* seedlings. (**b**) The reduced percentage of qP values before and after the transient addition of UV-A light (λ_max_ 365 nm, 2.48 μmol·m^−2^·s^−1^) with/without treatment of N-CD-3 in *M. hupehensis* seedlings.

Based on these above results, it could be concluded that the sites affected by N-CD-3 were probably located between PSII and PSI, that is, the Cyt *b_6_f* and PQs. Considering the precise structure of Cyt *b_6_f* protein complexes, N-CDs could hardly dock precisely with them internally and undergo redox reactions, thus the mobile electron transport carrier PQ was considered [40–43]. In the photosynthesis process, PSII oxidizes water and transfers the obtained electrons to PQs in the PQ pool [10]. Based on the redox characteristics of PQ-9 [11, 12] and the electron-donating capacity of CDs [31, 32], it is reasonable to expect that, similar to PSII, light-excited N-CD-3 transfers electrons to compound PQ-9 to generate the reduced PQH_2_-9, complementing electron flow in the PETC. After N-CD-3 is embedded in the thylakoid membrane, the contact and interplay between N-CD-3 and PQs are likely to take place with high probability (Fig. S13, see online supplementary material). The size of the PQ pools in the PETC is strictly conserved among different plant species [14]. Here, the limitations of the PQ pool were measured using the photochemical quenching parameter (qP) and the effective quantum yield of PSII photochemistry (Y(II)) to illustrate the electron transfer between N-CD-3 and PQ, in which Y(II) indicates the light energy utilization of PSII under steady-state conditions and qP reflects the oxidation state of plastoquinone Q_A_ in the photoreaction. Under the activation light illumination, the photosystem reached a steady state with relatively stable Y(II) values, and PQ-9 and PQH_2_-9 reached redox equilibrium. As expected, when UV-A light was supplemented, there was an obvious decrease in the Y(II) and qP values of the N-CD-3-treated leaves relative to the control (Fig. 4; FigsS15–S18, see online supplementary material), indicating the prior PQ redox equilibrium was disturbed by light-excited N-CD-3 and a new equilibrium was created. These shifts can be ascribed to the influence of photo-generated electrons originating from UV-A light-excited N-CD-3, which were transferred to the PQ-9 compounds in the PQ pool to generate PQH_2_-9. That is, because of the limitations of the PQ pool, a portion of PQ-9 compounds were reduced by accepting N-CD-3-transferred electrons, and the portion of PQ-9 compounds that accepted electrons from the PSII decreased, resulting in enhanced chlorophyll fluorescence, reduced oxidation of Q_A_, and lower Y(II) yield. Considering the PQ-9 role in the N-CD-3-involved electron transfer process, the effect of N-CD-3-involved electron transfer would decrease by enhancing light intensity, as more PQ were reduced by the photosynthetic system under high light intensity. As expected, the N-CD-3-induced improvement of seedling growth was weakened with the increase of light intensity (Fig. S19, see online supplementary material), providing further support for our hypothesis.

**Figure 5:**
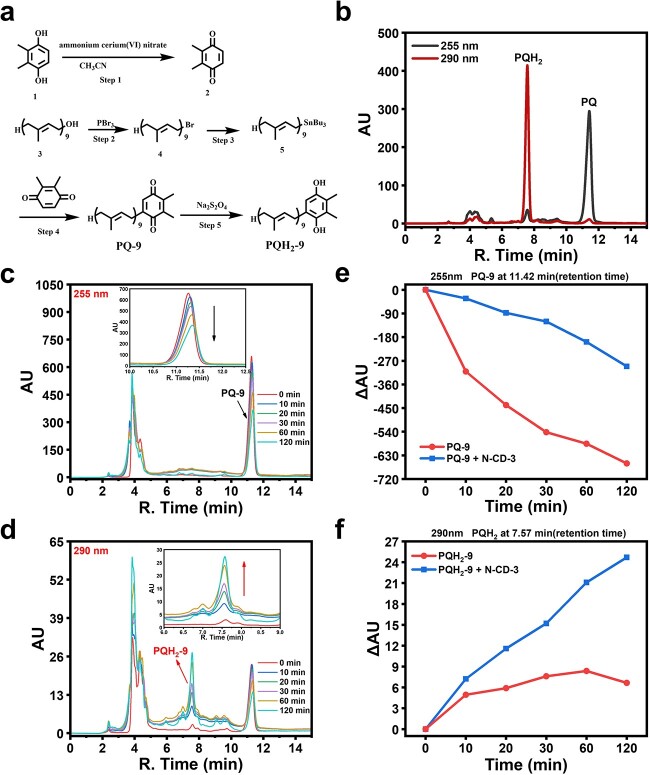
Electron transfer process from light-excited N-CD-3 to PQ-9* in vitro*. (**a**) Schematic of the PQ-9 synthesis route. (**b**) The retention time of PQ-9 and PQH_2_-9 in the HPLC spectra. (**c**–**d**) Absorbances of PQ-9 (255 nm, 11.42 min) and PQH_2_-9 (290 nm, 7.57 min) after UV-A light (λ_max_ 365 nm, 2.48 μmol·m^−2^·s^−1^) illumination in THF/H_2_O (V:V = 2:1) solution in the presence of N-CD-3 (2.0 g·L^−1^) and PQ-9 (0.2 g·L^−1^). (**e**–**f**) Absorbance in PQ-9 and PQH_2_-9 over time under 365 nm light irradiation (2.48 μmol·m^−2^·s^−1^) in the presence or absence of N-CD-3.

To further demonstrate the role of PQ-9 in the electron transfer process, the synthetic routine of the PQ-9 compound was rationally established (Fig. 5a). PQ-9 was successfully synthesized using 2,3-dimethylbenzene-1,4-diol and solanesol as raw materials, and then characterized using nuclear magnetic resonance (NMR) and high-resolution mass spectrometry (HRMS) (Figs S20–S21, see online supplementary material) [44]. To determine the electron transfer process from N-CD-3 to PQ-9, high-performance liquid chromatography (HPLC) was employed, which showed peaks at 7.57 min (290 nm wavelength) and 11.42 min (255 nm wavelength) retention times that belonged to PQH_2_-9 and PQ-9, respectively (Fig. 5b). Under UV-A light irradiation without N-CD-3, PQ-9 was consumed quickly, but almost no PQH_2_-9 was generated (Fig. S22, see online supplementary material). Interestingly, in the presence of N-CD-3, the peak at 11.42 min weakened while the peak at 7.57 min increased, proving that PQ-9 accepts electrons from the light-excited N-CD-3 to produce PQH_2_-9 (Fig. 5c–f). Taken together, via electron transfer from light-excited N-CD-3 to PQ-9, N-CD-3 may complement the PETC to enhance photosynthetic efficiency.

## Discussion

Photosynthesis directly or indirectly contributes to over 90% of the global agricultural yield. In recent research, plant nanobionics approaches have attracted increasing attention as high-potential pathways for photosynthesis augmentation [15, 19]. In this work, CDs with different nitrogen doping ratios were prepared by the hydrothermal method using citric acid and diethylenetriamine as raw materials, among which N-CD-3 exhibited excellent capacity to improve plant growth and enhance photosynthesis (Fig. 2). In particular, N-CD-3 also showed positive effects on orchard apple quality, where it was effective in increasing the Pn value of the tree and ultimately inducing redder fruits (14.36%) and higher sugar content (11.43%). This also lays the foundation for nanomaterials in apple production and demonstrates the great potential to improve fruit quality.

**Figure 6:**
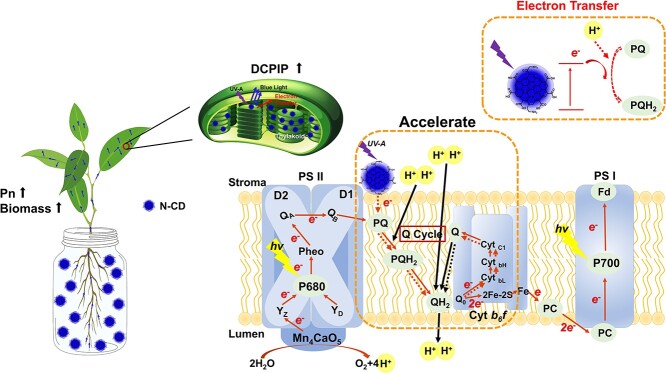
Hypothetical model for N-CDs-induced photosynthetic efficiency enhancement.

Recently, nanomaterials have emerged as an important player in agricultural biotechnologies [20, 28, 29, 31, 45]. While several studies on nanomaterial-modulated photosynthetic efficiency enhancements have been published [15, 16], the mechanisms by which nanomaterials interact with the photosynthesis system are relatively unknown, which hinders the development of agricultural nanomaterials. Three main possible mechanisms have been proposed, including light conversion [16, 22], FRET [21], and electron transfer [15]. In this work, we demonstrated that electron transfer from light-excited N-CDs to PQ-9, at least partially, augments photosynthetic efficiency. Interestingly, N-CD-3 partially relieved the photosynthesis defects of *mterf5* mutants with defective PSII function [37], but had little effect on *psa3* mutants with defective PSI function (Fig. 3d) [38, 39]. These results suggested that the photosystem components that N-CD-3 targeted are probably located between PSII and PSI, that is, the Cyt *b_6_f* and PQs. Furthermore, our comparative analysis of the HPLC of N-CD-3 + PQ-9 mixtures under light excitation indicated that PQ-9 can accept electrons from light-excited N-CD-3 to generate PQH_2_-9 (Fig. 5c–f). Therefore, a PQ-9-involved electron transfer mechanism for N-CD-3-induced photosynthetic efficiency enhancement was proposed, which is depicted in Fig. 6. N-CDs can be translocated from growth media throughout the plant body along a bottom-to-top pathway, with a portion finally reaching the thylakoid membrane of the photosynthesis centers in chloroplasts. Under UV-A light excitation, electron–hole pairs are generated in the N-CD-3, and then the photo-generated electrons are transferred to PQ-9 to produce PQH_2_-9, effectively complementing the PETC to enhance photosynthesis. Notably, considering the high conservation of photosystems, especially PQ pools [14], the N-CDs-induced photosynthesis enhancement is likely applicable to various plant species in addition to *A. thaliana* seedlings and apple trees used in this work.

Meantime, this work revealed the PQ-9-involved electron transfer mechanism by which nanomaterials improved photosynthesis. Based on this mechanism, more efficient nanomaterials for enhancing photosynthetic efficiency may be rationally designed by fine-tuning the starting materials and element doping in nanomaterials. Nanomaterials modulated with excellent electron-donating capacities and availability to photosystems have great potential for enhancing photosynthesis. It is worth noting that, besides the electron transfer process, N-CDs-induced light conversion and FRET processes probably also contributed to the photosynthesis enhancement, but further research measuring their contributions is required to confirm this supposition.

Furthermore, taking PQ-9 as the research subject, direct insight was obtained into both fundamental photosynthetic processes and nanomaterial-induced photosynthesis enhancement by identifying a pathway that circumvented interference by a complicated and variable intracellular environment. However, to obtain PQ-9 compounds at the microgram scale, the preparative HPLC technique is usually required, which is time-consuming and expensive [46, 47]. In this work, milligram-scale PQ-9 compound with high purity was easily obtained by chemical synthesis, a process that would be feasible even at the gram scale. The obtained PQ-9 was characterized by NMR and HRMS techniques. Inspiringly, isotope-labeled PQ compounds could also be synthesized with the introduction of C or O isotopes. These results provide a simple alternative strategy/technique for PQ-involved photosynthesis research.

## Conclusion

In conclusion, a novel model for N-CD-3-induced photosynthetic efficiency enhancement was proposed (Fig. 6). We revealed that N-CDs significantly enhanced photosynthetic efficiency, at least partially, via their influence on the electron transfer pathway, and PQ-9 serves as the electron-acceptor, providing a complement of the rate-limiting PETC to promote photosynthesis and apple fruit quality. Taken together, our work provides important insight into the electron transfer mechanism of nanomaterial-induced photosynthesis enhancement, and considering the high conservation of PETC among plant species, it informs the rational design of efficient nanomaterials for wide horticultural/agricultural applications.

## Materials and methods

### N-CDs synthesis

The N-CDs were synthesized via a one-pot hydrothermal method using citric acid (CA) as the carbon source and diethylenetriamine (DETA) as the nitrogen source. In brief, CA (250 mM) and DETA (25 mM, 125 mM, 200 mM, 250 mM, or 750 mM) were dissolved in 20 mL ultrapure water and then the solution was heated at 180°C for 4 h in a 100 mL Teflon-lined stainless steel autoclave. After cooling down, the obtained solution was filtered with 0.22 μm filter membrane to remove large particles. These N-CDs were further purified with acetone after moisture evaporation by rotary evaporator. Finally, according to the order of DETA dosage, these N-CDs were named as N-CD-1, N-CD-2, N-CD-3, N-CD-4, N-CD-5, respectively.

### N-CDs characterization

The JEM-2100F (JEOL, Tokyo, Japan) TEM was applied to observe N-CDs morphology, and the Nano Measurer software was used to measure the particle sizes. The heights of the N-CDs were detected using AFM (Bruker, Billerica, Massachusetts, USA) and the results were analysed by JPKSPM Data Processing software. Bio-TEM (HITACH, Tokyo, Japan) was used to observe the N-CD-3 location inside the chloroplast. The component and surface functional groups of CDs were analysed by XPS (Thermo, Waltham, Massachusetts, USA) and FT-IR (Thermo, Waltham, Massachusetts, USA). The UV–Vis absorption and photoluminescence spectra were measured on ultraviolet–visible absorption spectrophotometer (UV–Vis, Shimadzu, Kyoto, Japan) and fluorescence spectrophotometer (LUMINA, Thermo, Waltham, Massachusetts, USA), respectively. The zeta potential of N-CDs were measured by nanoparticle size analyser (Zetasizer Nano ZS90, Malvern, Malvern, England).

### Isolation of chloroplast

Chloroplasts were extracted according to the previous method [15]. Chloroplast concentration was referred to the total chlorophyll concentration, which was measured according to Arnon’s method [47]. The chlorophyll concentration: (mg·mL^−1^) = OD_652_ × 50/34.5.

### Preparation of N-CDs + chloroplast complex

Initially, the mixture of chloroplast suspensions (100 mg·L^−1^) and N-CDs (300 mg·L^−1^) was incubated in the dark at 4°C for 2 h and then the generated N-CDs + chloroplast composites were collected by centrifugation at 3000 rpm for 3 min at 4°C, and used for further characteristics. Furthermore, the absorption spectra and fluorescence spectra of these composites were measured using UV–Vis (Shimadzu, Kyoto, Japan) and fluorescence spectrophotometer (LUMINA, Thermo, Waltham, Massachusetts, USA), respectively.

### Plant materials and growth conditions

#### A. thaliana

After sterilizing with 75% ethanol and 2% sodium hypochlorite, the seeds of *A. thaliana* wild-type Col-0, *mterf5* mutant and *psa3* mutant were sowed onto 1/2 MS medium, vernalized at 4°C for 96 h under dark conditions, and transferred to light conditions. Then, they were cultured for 3 d in an illumination incubator (22°C, 16 h/8 h light/dark). Subsequently, (i) the Col-0 seedlings were transferred to 1/2 MS medium (1% sucrose) with different N-CDs (300 mg·L^−1^) and cultured for 14 d under light illumination (20.43 μmol·m^−2^·s^−1^) with UV-A light (λ_max_ 365 nm, 0.83 μmol·m^−2^·s^−1^) (Fig. S9, see online supplementary material); (ii) the Col-0, *mterf5*, and *psa3* seedlings were transferred to 1/2 MS medium (0% sucrose) with N-CD-3 (300 mg·L^−1^) and cultured for 30 d under light illumination (20.43 μmol·m^−2^·s^−1^) with UV-A light (λ_max_ 365 nm, 0.83 μmol·m^−2^·s^−1^) (Fig. 3d); (iii) the Col-0 and *mterf5* seedlings were transferred to 1/2 MS medium (1% sucrose) with N-CD-3 (300 mg·L^−1^) and cultured for 14 d under light illumination (20.43 μmol·m^−2^·s^−1^) with UV-A light (λ_max_ 365 nm, 0.83 μmol·m^−2^·s^−1^) (Fig. S14, see online supplementary material); and (iv) the Col-0 seedlings were transferred to 1/2 MS medium (1% sucrose) with N-CD-3 (300 mg·L^−1^) and cultured for 10 d under low light (20 μmol·m^−2^·s^−1^)/middle light (100 μmol·m^−2^·s^−1^)/high light (350 μmol·m^−2^·s^−1^) containing UV-A light (λ_max_ 365 nm, 0.83 μmol·m^−2^·s^−1^) (Fig. S19, see online supplementary material). The light conditions used in the experiments can be checked in (Fig. S23, see online supplementary material).

#### M. hupehensis

After 3 d of soaking, the seeds were laminated at 4°C for 1 ~ 2 months under dark conditions. Then, the *M. hupehensis* seeds were sowed on soil (vermiculite: soil matrix = 1:2). After the appropriate 2 weeks, seedlings with two true leaves were transplanted into Hoagland nutrient solution for hydroponic culture. After adaptation for 2 d, the seedlings with consistent growth were transferred to Hoagland nutrient solution with 300 mg·L^−1^ N-CDs [36]. All treatments were cultured under light illumination (20.43 μmol·m^−2^·s^−1^) with UV-A light (λ_max_ 365 nm, 0.83 μmol·m^−2^·s^−1^) for 30 d to test related indicators (Fig. S23, see online supplementary material). After treatment with N-CDs for 2 d, several seedlings were used to determine the distribution and localization of N-CDs by observing the thin slices of different parts of seedlings with a high-resolution confocal laser microscope (LSM 880, Zeiss, Oberkochen, Germany) (408 nm excitation, 410–528 nm and 531–703 nm collection, respectively).

#### *Gala/M9* apple tree

The field trial was conducted at the apple demonstration garden in Yiyuan County, Shandong Province, China (from 28 April 2023 to 20 August 2023). Eight-year-old *Gala/M9* apple trees were selected for the experiment, and six trees of neighboring growth status and location were chosen as replicates in each group. Apple trees were sprayed every 15 d for 7 times with water as the control group and N-CD-3 (1000 mg·L^−1^) as the treatment group. The Pn values of leaves were measured in four directions at the same height, and each direction was measured three times; apple fruits were harvested in four directions at the same height, and three apple fruits were randomly picked in each direction for subsequent physiological index measurements.

### Measurement of plant physiology and growth

After treatment for some time, the growth performances and related physiological indexes of plants in the treatment group and control group were measured. The single plant fresh weight, root length, stem length, leaf area, and fruit shape index were measured in each treatment. The chlorophyll contents were determined by ethanol method [49]. The soluble sugar content was determined by phenol method. The anthocyanin content was measured by methanol-HCl method [50]. The soluble solids content of fruits was determined by saccharimeter (TY/HTPTD-45, Tuya, Huizhou, China). Stomatal aperture assays were performed as previously described [51]. The Pn was measured by Ciras-3 Portable Photosynthetic Fluorometry System (PP Systems, Haverhill, Massachusetts, USA). Fruit color, luster, and hardness were determined by colorimeter (NR60CP+, 3nh, Shenzhen, China), luster meter (NHG268, 3nh, Shenzhen, China), and texture analyser (TA. XT plus mass spectrometer, Stable Micro Systems, Godalming, England), respectively.

### Hill reaction

Hill reaction was conducted to explore the effect of N-CDs on the photosynthetic activity of chloroplasts in terms of the reduction rate of DCPIP [15]. To determine the saturating light of isolated chloroplasts, the reduction of 2,6-dichlorophenolindophenol (DCPIP, 500 μM) by isolated chloroplasts was investigated at different light intensities (27.38, 54.80, 82.30, 109.51, 123.23, 136.95, 150.62, 164.31, 191.70, and 219.08 μmol·m^−2^·s^−1^). To investigate the effect of N-CDs on the photosynthetic activity of chloroplasts, the absorbance values variation at 600 nm of DCPIP (500 μM), N-CDs (300 mg·L^−1^) + DCPIP, chloroplasts (100 mg·L^−1^) + DCPIP, inactivated chloroplasts + DCPIP and chloroplasts + N-CDs + DCPIP were determined under saturated light (150.62 μmol·m^−2^·s^−1^) containing UV-A (λ_max_ 365 nm, 4.06 μmol·m^−2^·s^−1^). The OD_600_ was recorded at a 1-minute interval using UV–Vis (Shimadzu, Kyoto, Japan). The calculation formula:

DCPIP Reduction(ΔA) = A_n_-A_0_.

Reduced DCPIP(%) = (A_0_-A_n_)/(A_0_-A_24_).

A_n_ represents the OD_600_ value after n min of light exposure, A_0_ represents the OD_600_ value before light exposure, A_24_ represents the OD_600_ value after 24 min of light exposure.

### Synthesis of PQ-9

The synthesis route of PQ-9 was depicted in Fig. 5a, which was designed according to the reported method [44] with some modifications. Detailed synthesis steps can be found in the supplementary data. Then, the obtained PQ-9 was characterized by NMR (Bruker, Billerica, Massachusetts, USA) and HRMS (Thermo, Waltham, Massachusetts, USA) (Figs S20–S21, see online supplementary material).

### Analysis of electron transfer from N-CDs to PQ *in vitro*

Subsequently, the HPLC (Shimadzu, Kyoto, Japan) was used to determine the retention time of PQ-9 and PQH_2_-9 (Fig. 5b). The chromatographic conditions were determined as follows: Hypersil GOLD aQ C^18^ column 4.6 mm_*_250 mm 5 μm (Thermo, Waltham, Massachusetts, USA), mobile phase 50% ethanol and 50% acetonitrile, flow rate 1 mL·min^−1^. The PQ-9 and PQH_2_-9 were dissolved in the solvent (tetrahydrofuran and water =2:1) to test their characteristic wavelength and retention time. Subsequently, the PQ-9 solution (0.3 mg·mL^−1^, tetrahydrofuran) was mixed with N-CD-3 (6 mg·mL^−1^) in a ratio of 2:1, and irradiated under UV-A (λ_max_ 365 nm, 2.48 μmol·m^−2^·s^−1^) irradiation. After irradiation for 0, 10, 20, 30, 60, and 120 min, the substance changes of PQ-9 were analysed by HPLC under the same experimental conditions. The calculation formula:

ΔAU = AU_n_-AU_0_.

AU_n_ represents the OD_255_/OD_290_ value after n min of UV-A irradiation, AU_0_ represents the OD_255_/OD_290_ value before UV-A irradiation.

### The measurement of qP and Y(II) values

The pool restriction of the PQ pool was used to illustrate the electron transfer relationship between N-CDs and PQ. Specifically, the photochemical quenching parameter (qP) and the actual photochemical efficiency (Y(II)) under light were detected using Dual-Pam 100 (WALZ, Evfeltrich, Germany) for the control and N-CD-3-treated groups. Firstly, the program was set as fluo+P700 slow phase mode. Meantime, the measurement parameters were set as follows: the intensity of Fluo Meas. Light: 20; the intensity of P700 Meas. Light: 10; the intensity of Act. Red Light: 6; the intensity of Far Red Light: 18; the intensity of Act. Blue Light: 8; the intensity of Sat. Pulse Light: 8. Subsequently, the script was modified based on Ind. Curve, number of detection points was increased from the original saturated pulsed light detection points. Then, the leaves of *M. hupehensis* were placed between the two detectors with an additional UV-A light source (λ_max_ 365 nm, 2.48 μmol·m^−2^·s^−1^) near the detectors. Finally, running the modified script program, when the Y(II) or qP value was stabilized, UV-A light (λ_max_ 365 nm, 2.48 μmol·m^−2^·s^−1^) was added, and the program was terminated when the Y(II) or qP value stabilized again. The calculation formula:

Y(II) Reduced Percentage (%) = (Y(II)_(before UV-A illumination)_ –Y(II)_(after the UV-A illumination)_) / Y(II)_(before UV-A illumination)_ × 100%.

Y(II)_(before UV-A illumination)_ represents the stable Y(II) value before UV-A illumination, Y(II)_(after UV-A illumination)_ represents the stable Y(II) value after UV-A illumination.

qP Reduced Percentage (%) = (qP_(before UV-A illumination)_ – qP_(after the UV-A illumination)_) / qP_(before UV-A illumination)_ × 100%.

qP_(before UV-A illumination)_ represents the stable qP value before UV-A illumination, qP_(after UV-A illumination)_ represents the stable qP value after UV-A illumination.

### Statistical analysis

All the treatments were repeated more than three times in biology and technology. Values presented in this manuscript were expressed as means ± standard deviation (SD). Statistical significance of all data was determined using a one-way analysis of variance (ANOVA) and compared using Tukey and LSD test at *P* < 0.05 levels in DPS7.05.

## Supplementary Material

Web_Material_uhae016
