# Supplementary material for: Enhanced photosynthetic efficiency by nitrogen-doped carbon dots via plastoquinone-involved electron transfer in apple
Source: Hortic Res. 2024 Jan 12;11(3):uhae016. doi: 10.1093/hr/uhae016 (PMC10940122; doi:10.1093/hr/uhae016)
Supplement: Web_Material_uhae016 [file web_material_uhae016.docx]

**Supporting Information**

Enhanced photosynthetic efficiency by nitrogen-doped carbon dots via plastoquinone-involved electron transfer in plants

Xiuli Jing^1^, Yankai Liu^1^, Xuzhe Liu^2^, Yi Zhang^3^, Guanzhu Wang^1^, Fei Yang^1^, Yani Zhang^1^, Dayong Chang^4^, Zhen-Lu Zhang^1^, Chun-Xiang You^1,*^, Shuai Zhang^2,*^ and Xiao-Fei Wang^1,*^

^1^ Apple Technology Innovation Center of Shandong Province, Shandong Collaborative Innovation Center of Fruit & Vegetable Quality and Efficient Production, National Key Laboratory of Wheat Improvement, College of Horticulture Science and Engineering, Shandong Agricultural University, Taian, Shandong, China

^2^ Key Laboratory of Agricultural Film Application of Ministry of Agriculture and Rural Affairs, College of Chemistry and Material Science, Shandong Agricultural University, Taian, Shandong, China

^3^ College of Life Science, Shandong Agricultural University, Taian, Shandong, China;

^4^ Yantai Goodly Biotechnology Co., LTD., Yantai, Shandong, China

Author list.

Xiuli Jing: Email-1337625980@qq.com, Tel-15725998772;

Yankai Liu: Email-735833078@qq.com, Tel-15688823175;

Xuzhe Liu: Email-2250113106@qq.com, Tel- 17861500920;

Yi Zhang: Email-zhangyi@sdau.edu.cn, Tel-15650455582;

Guanzhu Wang: Email-1261954452@qq.com, Tel-15646705192;

Fei Yang: Email-15588543593@163.com, Tel-15588543593;

Yani Zhang: Email- 2243234057@qq.com, Tel-17662591296;

Dayong Chang: Email- ytgoodly@163.com, Tel-13562536876;

Zhen-Lu Zhang: Email-zzhenlu0526@sdau.edu.cn, Tel-18854337005.

*Corresponding author.

Xiao-Fei Wang: Email-wangxiaofei@sdau.edu.cn, Tel-13905388193;

Shuai Zhang: Email-hyzs@sdau.edu.cn, Tel-18553862388;

Chun-Xiang You: Email-youchunxiang@126.com, Tel-15069897490.

**Methods**

**Synthesis of Plastoquinone-9**

Step 1: 3-Dimethylcyclohexa-2,5-diene-1,4-dione (compound 2)

To a solution of 2,3-dimethylbenzene-1,4-diol (1.146 g, 8.29 mmol) in 20 mL solution (V_acetonitrile_: V_water_ = 1: 1) was added ammonium cerium(IV) nitrate (45.45 g, 82.9 mmol) and 2,6-pyridinedicarboxylic acid (10 equiv.). The mixture was stirred at 0 ^o^C and room temperature for 15 min, respectively. Subsequently, the mixture was transferred to a separatory funnel containing 20 mL water and 20 mL ether. The mixture was extracted three times (3 × 20 mL) with ether. The organic layer was gathered and dried over MgSO_4_. Finally, the solvent was removed under vacuum to obtain the target compound 2.

Step 2: Solanesyl Bromide (compound 4)

To a solution of 25 mL ether and 16 mL hexane was added solanesol (1.5756 g, 2.5 mmol) and 5 drops of pyridine. Then 675.8 mg (2.5 mmol) phosphorus tribromide was added dropwise under an ice bath. The mixture was stirred in ice bath for 1 h and at room temperature for 1 h. After the completion of the reaction, the mixture was poured out into a separatory funnel, which was charged with 50 mL water/ice and 50 mL petroleum ether. The mixture was extracted three times (50 mL ×3) with petroleum ether. The organic layer was combined and dried over Na_2_SO_4_. Finally, the solvent was removed under reduced pressure to obtain the crude product solanesyl bromide (4), which was used directly without further purification.

Step 3: Solanesyltributyltin (compound 5)

1.58 mL butyllithium in hexane (2.5 M) was injected via a syringe into 10 mL THF solution containing diisopropylamine (0.635 mL, 4.53 mmol) under ice-water bath. After 5 min, 0.80 mL tributyltin hydride (2.40 mmol) was added carefully. After stirring for 15 minutes, the mixture was cooled to -60 ^o^C under the liquid N_2_/acetone bath. Then, solanesyl bromide (1.93 g, 2.0 mmol) in 20 mL THF was added slowly, and the obtained mixture was warmed up slowly to -40°C for approximately 2 h. Subsequently, the mixture was quenched by 20 mL saturating NH_4_Cl solution. The mixture was extracted twice (2×30 mL) with ether, washed with saturating NaCl solution and dried over by Na_2_SO_4_. The target compound solanesyltributyltin (5) was obtained and used directly without further purification.

Step 4: Plastoquinone-9 (compound PQ-9)

92 mg (0.675 mmol) 3-dimethylcyclohexa-2,5-diene-1,4-dione (compound 2), 624.5 mg (2.695 mmol) silver oxide and 20 mL dichloromethane were added successively into a 100 mL round bottom flask. After the injection of 1.16 mL boron trifluoride-diethyl ether, the mixture was cooled to -80 ^o^C at dark. Then solanesyltributyltin (5) (1.23 g, 1.36 mmol) in 10 mL dichloromethane was added slowly. This mixture warmed up slowly to -20 ^o^C for about 2 h, and then 20 mL of saturated NaCl solution was added. After the completion of the reaction, the mixture was filtrated over diatomite. The mixture was extracted twice with dichloromethane (2×20 mL). The organic layer was gathered and dried with Na_2_SO_4_. Dichloromethane was removed under vacuum to obtain the crude product. Purification over silica with an eluant of 2% ethyl acetate /98% n-hexane yielded 0.065 g (0.087 mmol, 13%) of pure product PQ-9. ^1^H NMR (400 MHz, CDCl_3_) δ 6.46 (t, 1H), 5.11-5.09 (m, 2H), 3.13 (d, 2H, tail 1-CH_2_), 2.08-1.94 (m, 40H, tail 16 CH_2_, 2’-CH_3_, 3’-CH_3_) tail end trans CH_3_, tail 3-CH_3_), 1.68-1.56 (m, 30 H, tail cis 35’-CH_3_, tail 7 CH_3_, tail 3’-CH_3_, tail end trans 36-CH_3_). ^13^C NMR (101 MHz, CDCl_3_) δ 187.93, 187.79, 148.09, 141.09, 140.69, 139.82, 135.78, 135.55, 135.17, 135.09, 135.06, 135.03, 135.01, 132.15, 131.36, 124.55, 124.41, 124.34, 124.27, 123.95, 123.75, 118.27, 77.48, 77.16, 76.84, 39.88, 39.86, 39.83, 27.60, 26.90, 26.85, 26.81, 26.62, 25.83, 17.81, 16.27, 16.20, 16.16, 12.51, 12.17. HRMS (APCI), calcd for C_53_H_81_O_2_^+^ (M^+^H)^+^, 749.6231 found 749.6230; C_53_H_79_O_2_^-^ (M^-^H)^-^, 747.6086, found 747.6079.

Step 5: PQH_2_-9

A 50 mL flask was added compound PQ-9 (18.2 mg, 0.024 mmol) in 10 mL ether and 3.0 g Na_2_S_2_O_4_ in 10 mL 0.01 M NaOH. This mixture was vigorously stirred until the yellow color disappeared. And then the mixture was extracted twice with ether (2× 20 mL). The organic layers were gathered, washed with saturating NH_4_Cl solution and dried over Na_2_SO_4_. The target compound PQH_2_-9 was obtained after concentration under vacuum.

**Figures**


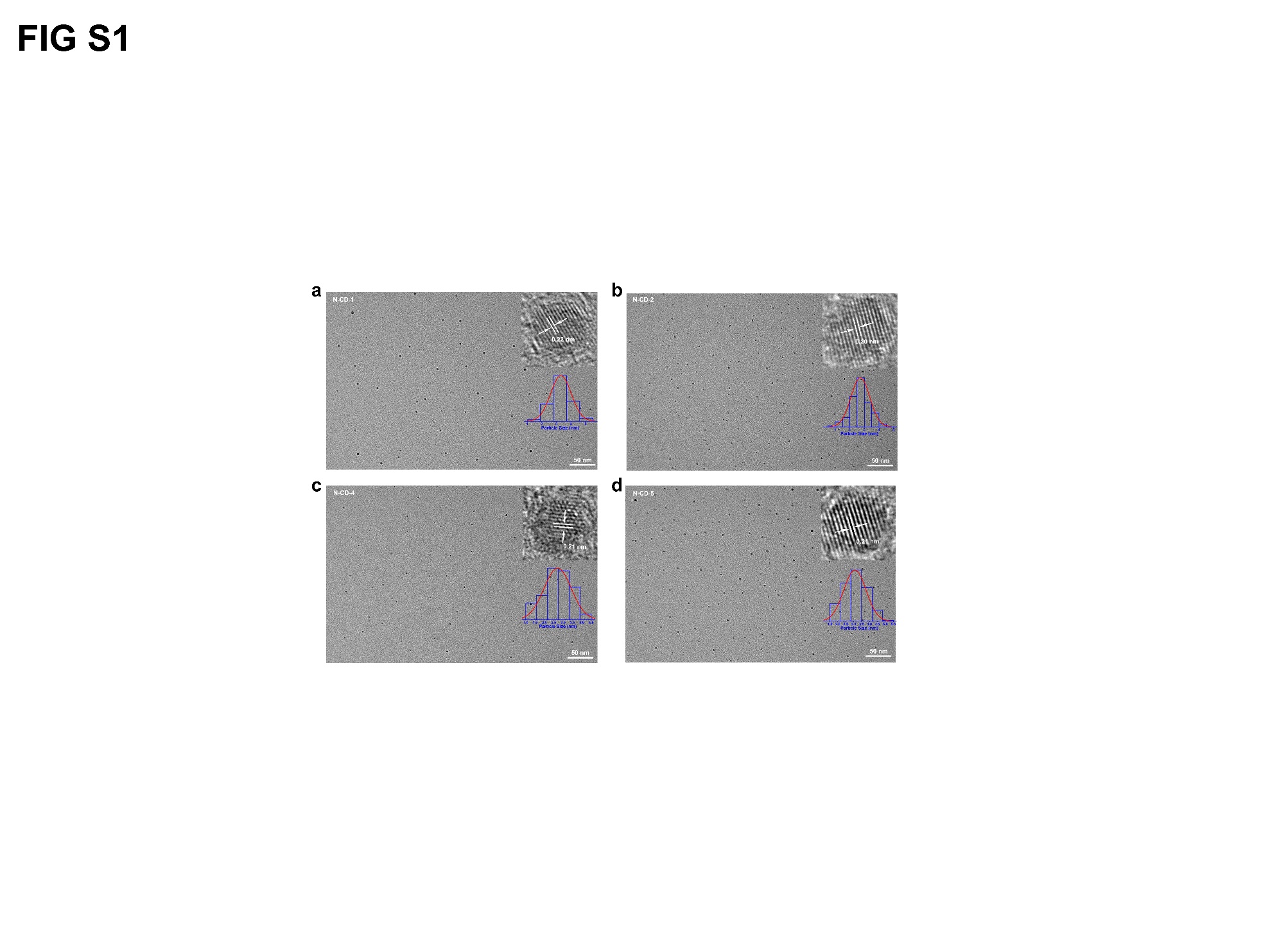


**Figure S1.** HRTEM images and the size distribution (inserted image) of N-CD-1/2/4/5.


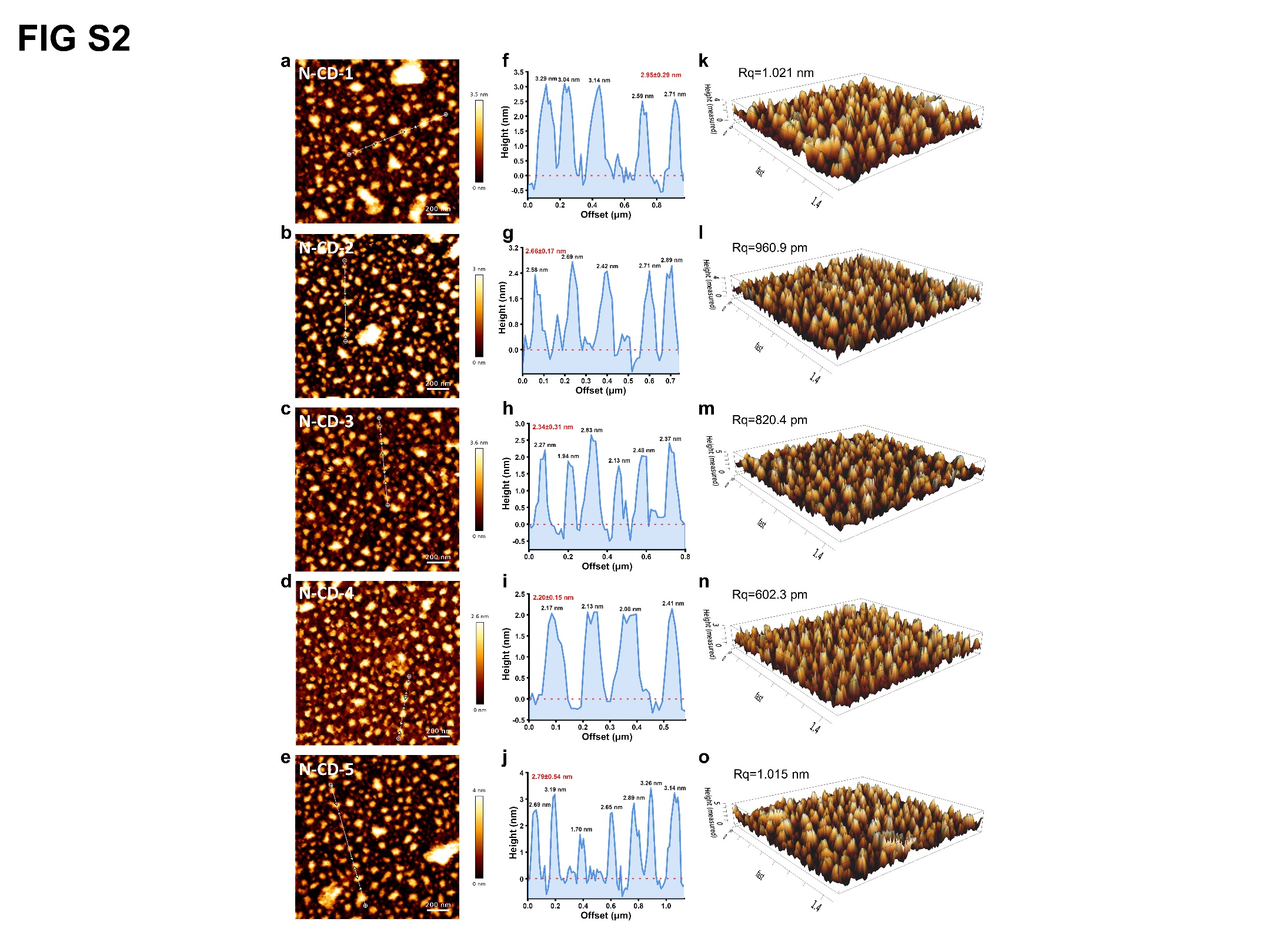


**Figure S2.** AFM characterization of N-CDs. (a-d) The 2D image of AFM of N-CDs. (f-j) The height analyses of N-CDs. (k-o) The 3D image of AFM of N-CDs.


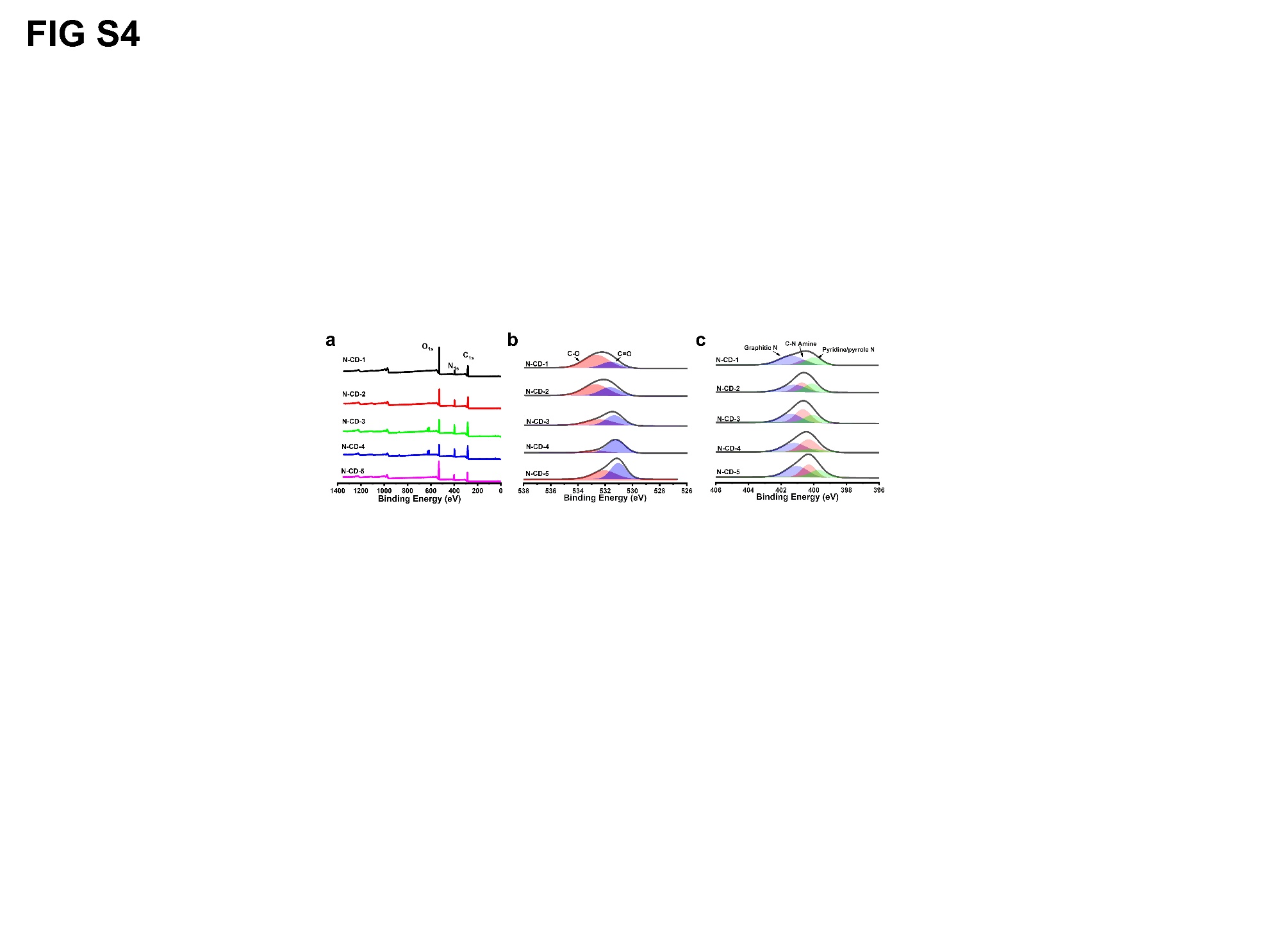


**Figure S3.** XPS characterization of N-CDs. (a) XPS full scan spectrum of N-CDs. (b-c) High-resolution XPS spectra of O_1s_ (b) and N_1s_ (c) of N-CDs.


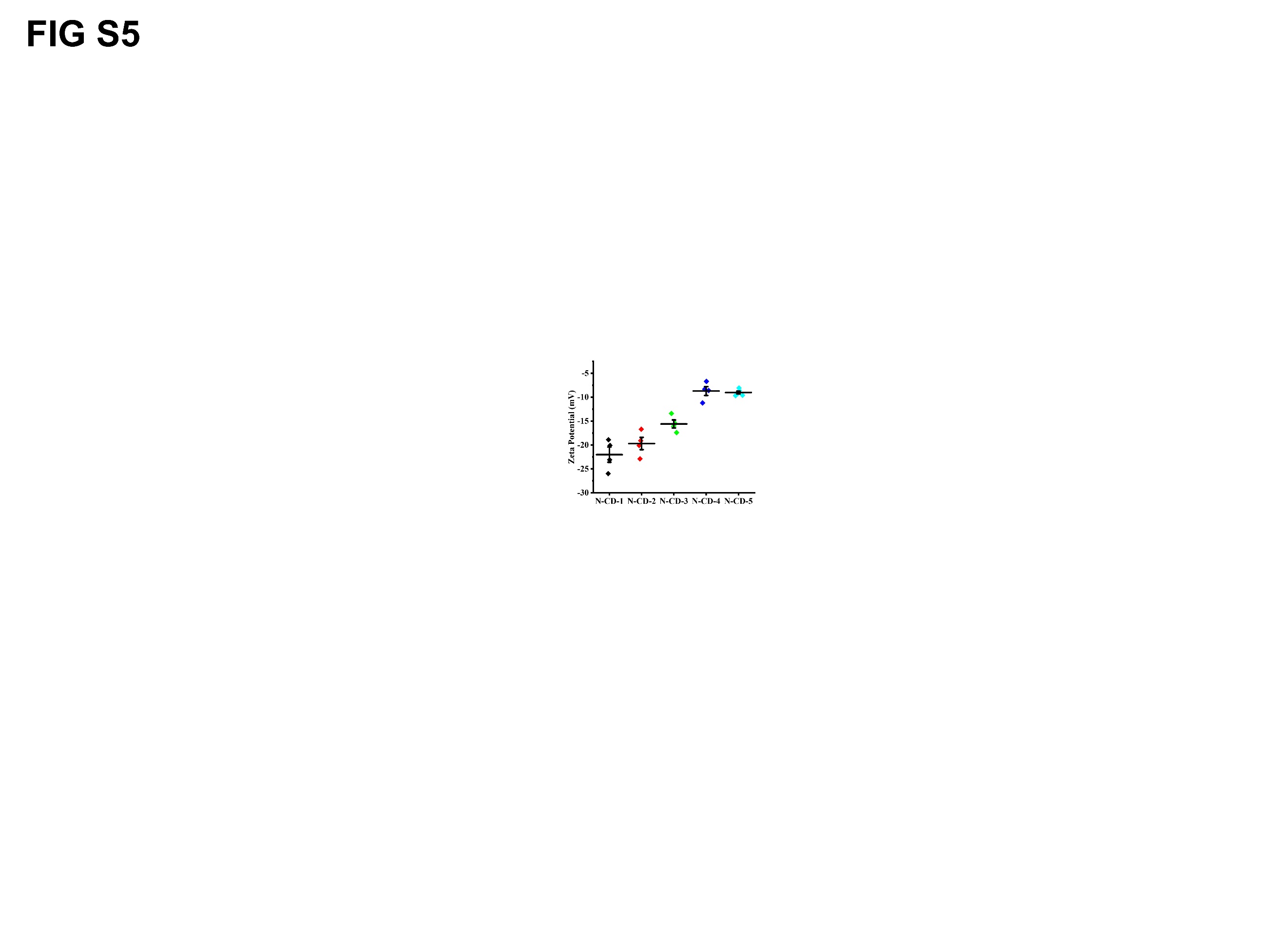


**Figure S4.** The zeta potential of N-CDs at pH 9 condition. Error bars denote SD.


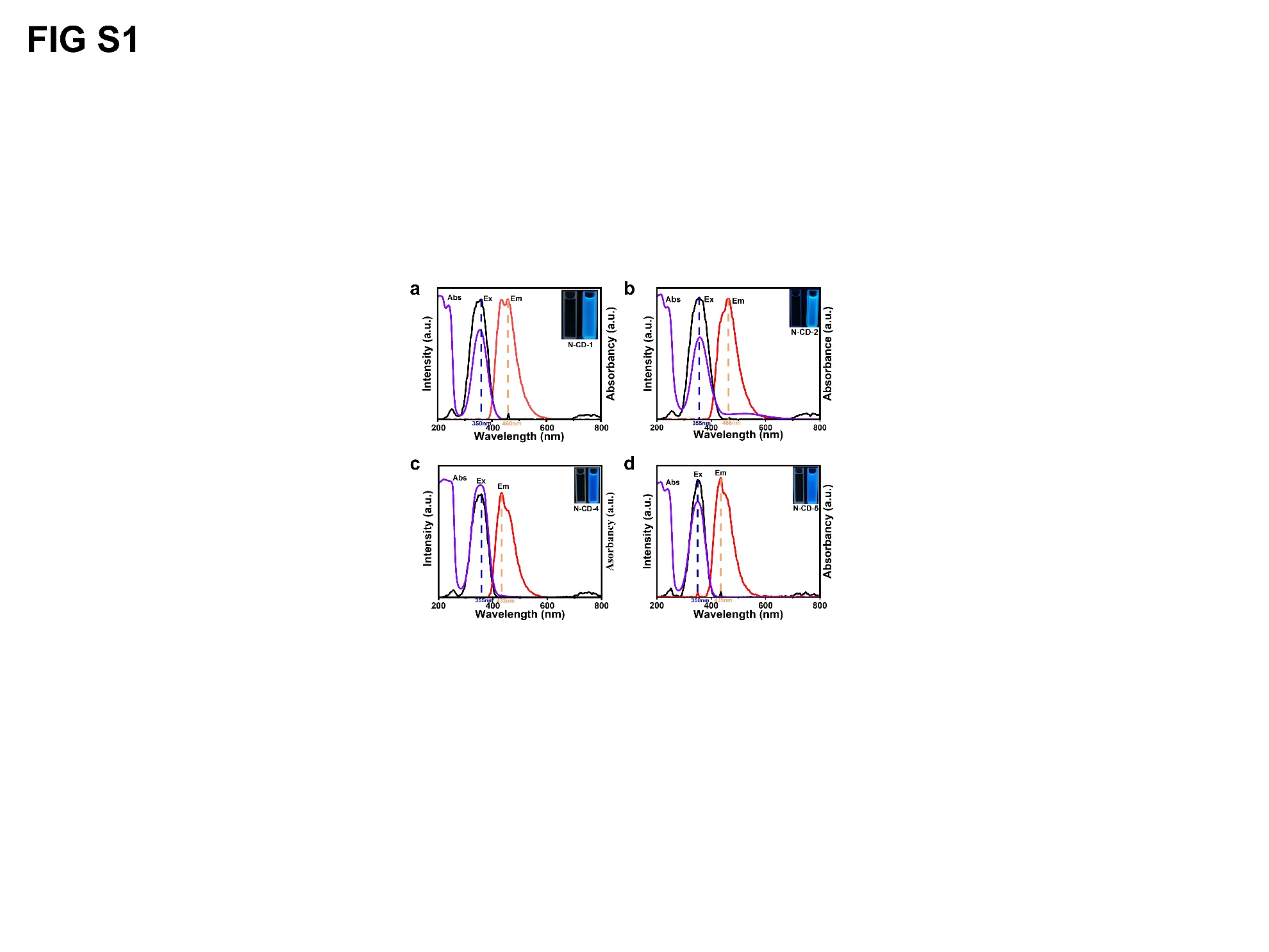


**Figure S5.** The UV-Vis absorption spectra (Abs, purple), excitation spectra (Ex, black) and emission spectra (Em, red) of N-CD-1 (a), N-CD-2 (b), N-CD-4 (c), N-CD-5 (d).


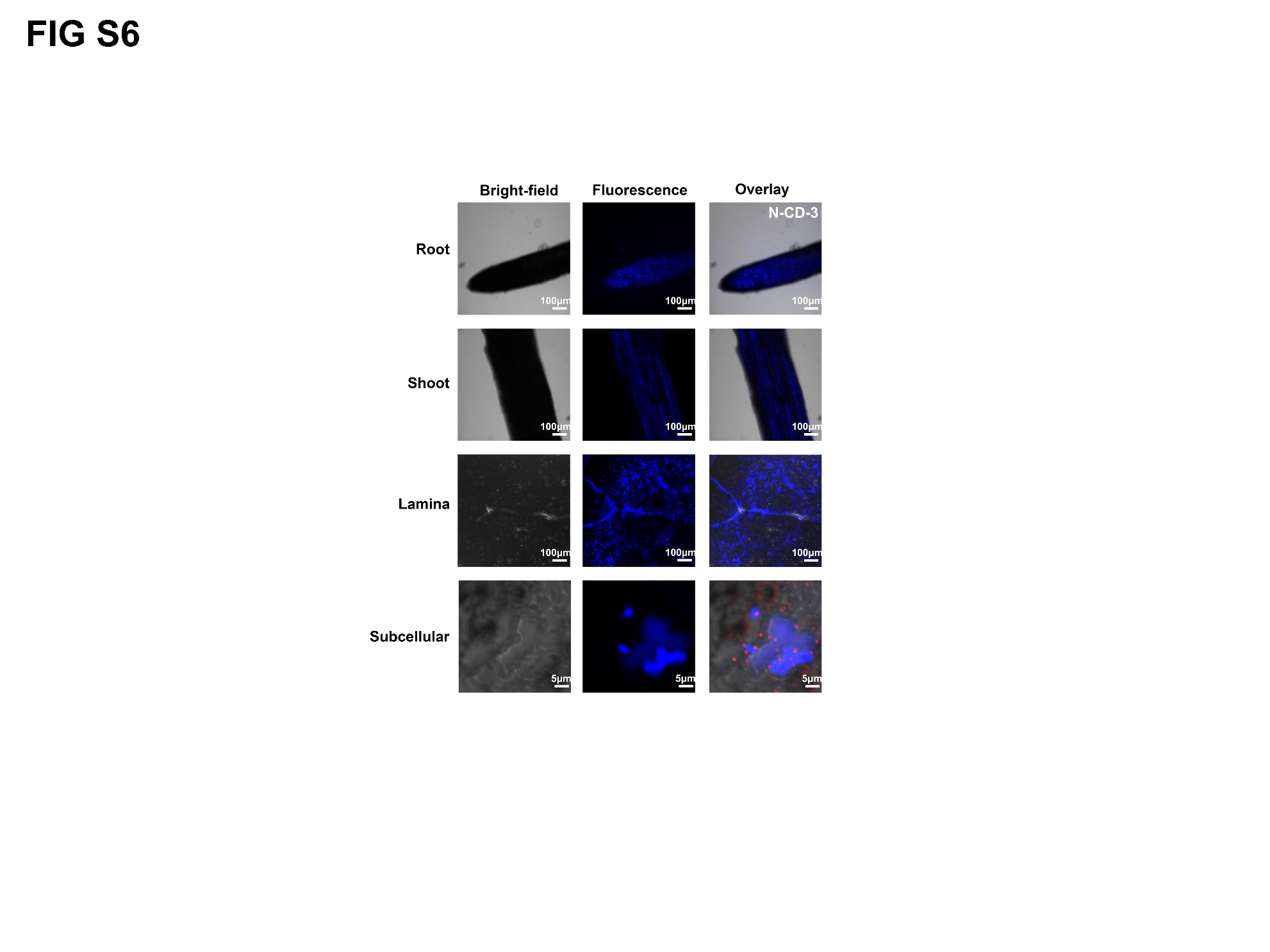


**Figure S6.** Confocal microscopy images of root, shoot, lamina and chloroplasts in *Malus hupehensis* seedlings with the treatment of N-CD-3 (300 mg·L^-1^). (408 nm excitation, 410 - 528 nm and 531 - 703 nm collection, respectively.)


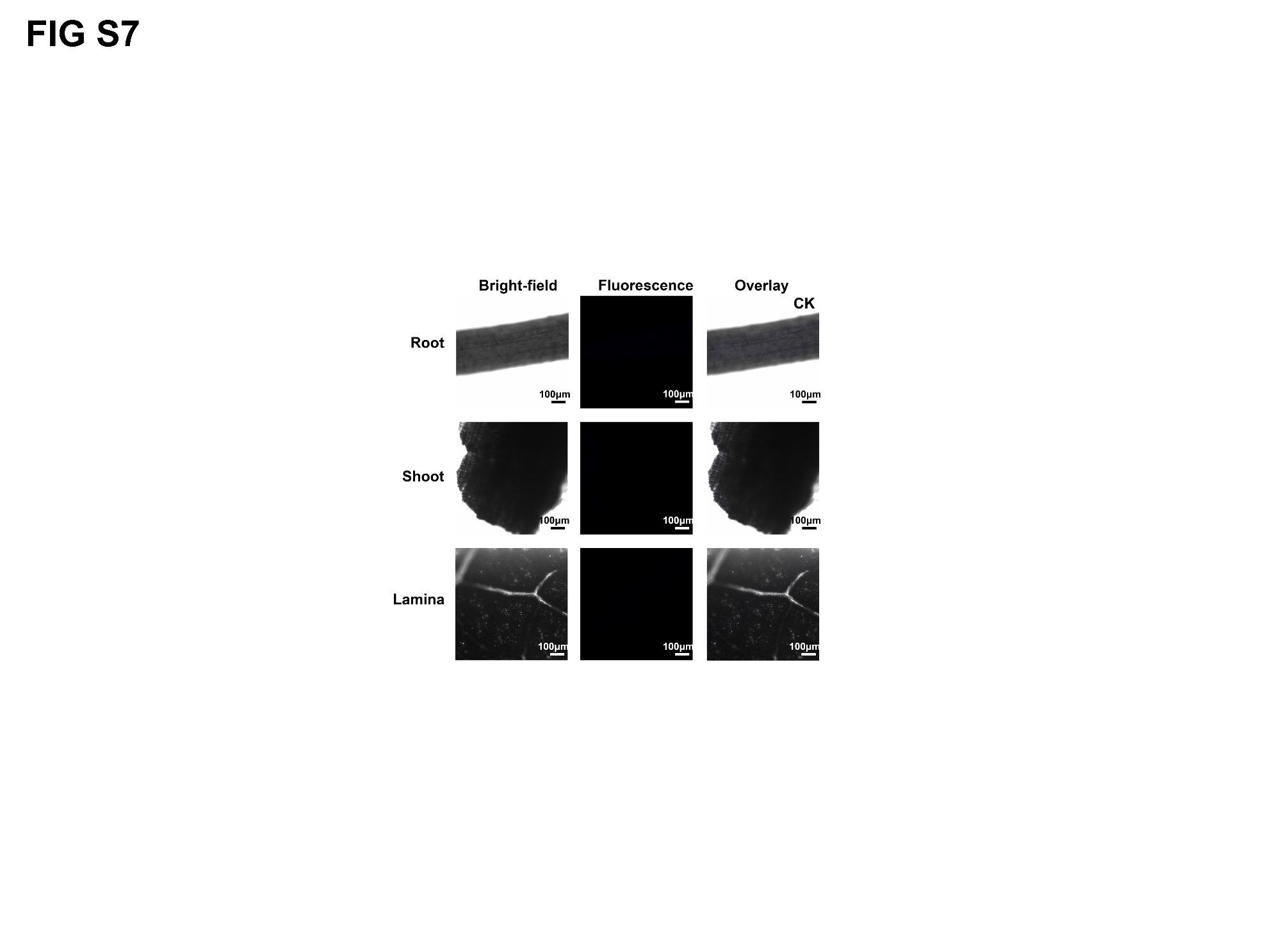


**Figure S7.** Confocal microscopy images of root, shoot and lamina in *Malus hupehensis* seedlings without the treatment of N-CDs. (408 nm excitation, 410 - 528 nm collection)


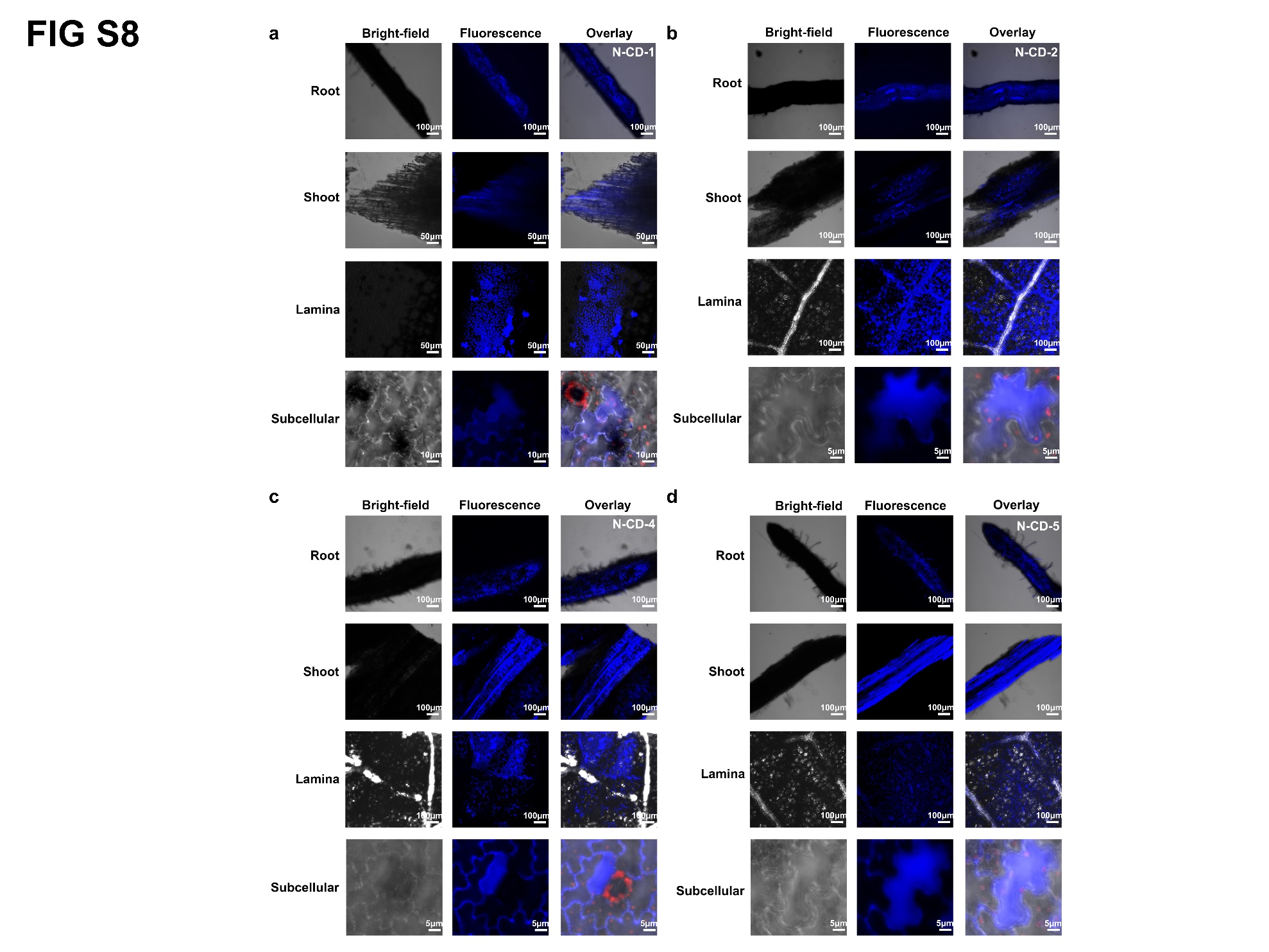


**Figure S8.** Confocal microscopy images of root, shoot, lamina and chloroplasts in *Malus hupehensis* seedlings treated with 300 mg·L^-1^ N-CD-1 (a), N-CD-2 (b), N-CD-4 (c), and N-CD-5 (d), respectively. (408 nm excitation, 410-528 nm and 531 - 703 nm collection, respectively)


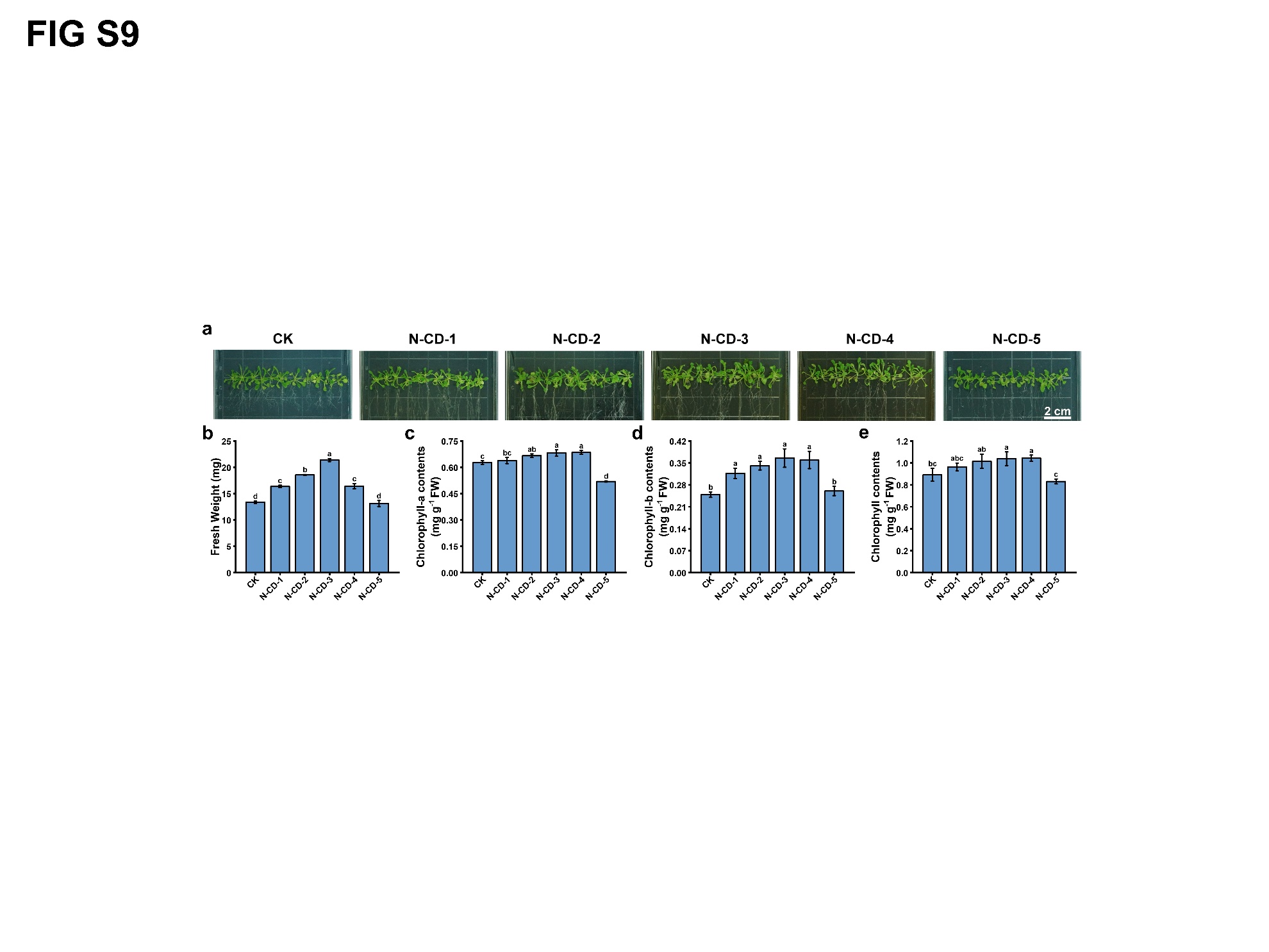


**Figure S9.** Effects of N-CDs on the *Arabidopsis thaliana* seedlings. (a) Effects of N-CDs (300 mg·L^-1^) on the seedlings growth under light illumination (20.43 μmol·m^-2^·s^-1^) containing UV-A (λ_max_ 365 nm, 0.83 μmol·m^-2^·s^-1^). Pictures captured after 14 d of cultivation. (b-e) The fresh weight (b), chlorophyll a content (c), chlorophyll b content (d), chlorophyll content (e) with/without the N-CDs treatment of seedlings. One-way analysis of variance (ANOVA) was used to analyze all data and error bars represent the SD from more than three biological replicates with three parallel experiments. Different lowercase letters represent significant differences (*P*<0.05, Tukey test).


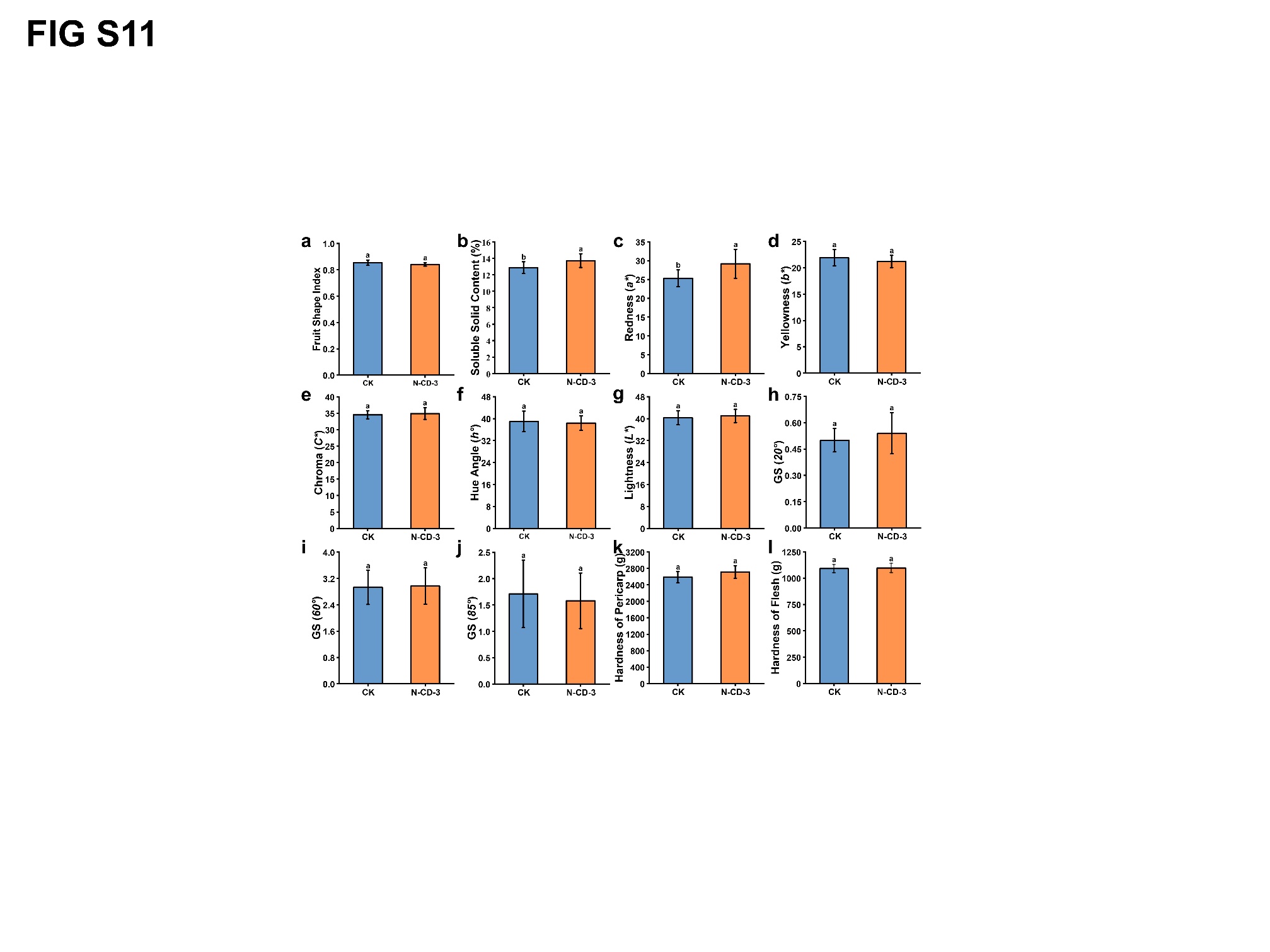


**Figure S10.** Effect of N-CD-3 on fruit quality of ‘*Gala/M9*’ apple. (a-b) Fruit shape index (a) and soluble solid content (b) with/without the N-CD-3 treatment. (c-g) The fruit color with/without the N-CD-3 treatment. (h-j) The fruit luster with/without the N-CD-3 treatment. (k-l) The fruit hardness with/without the N-CD-3 treatment.


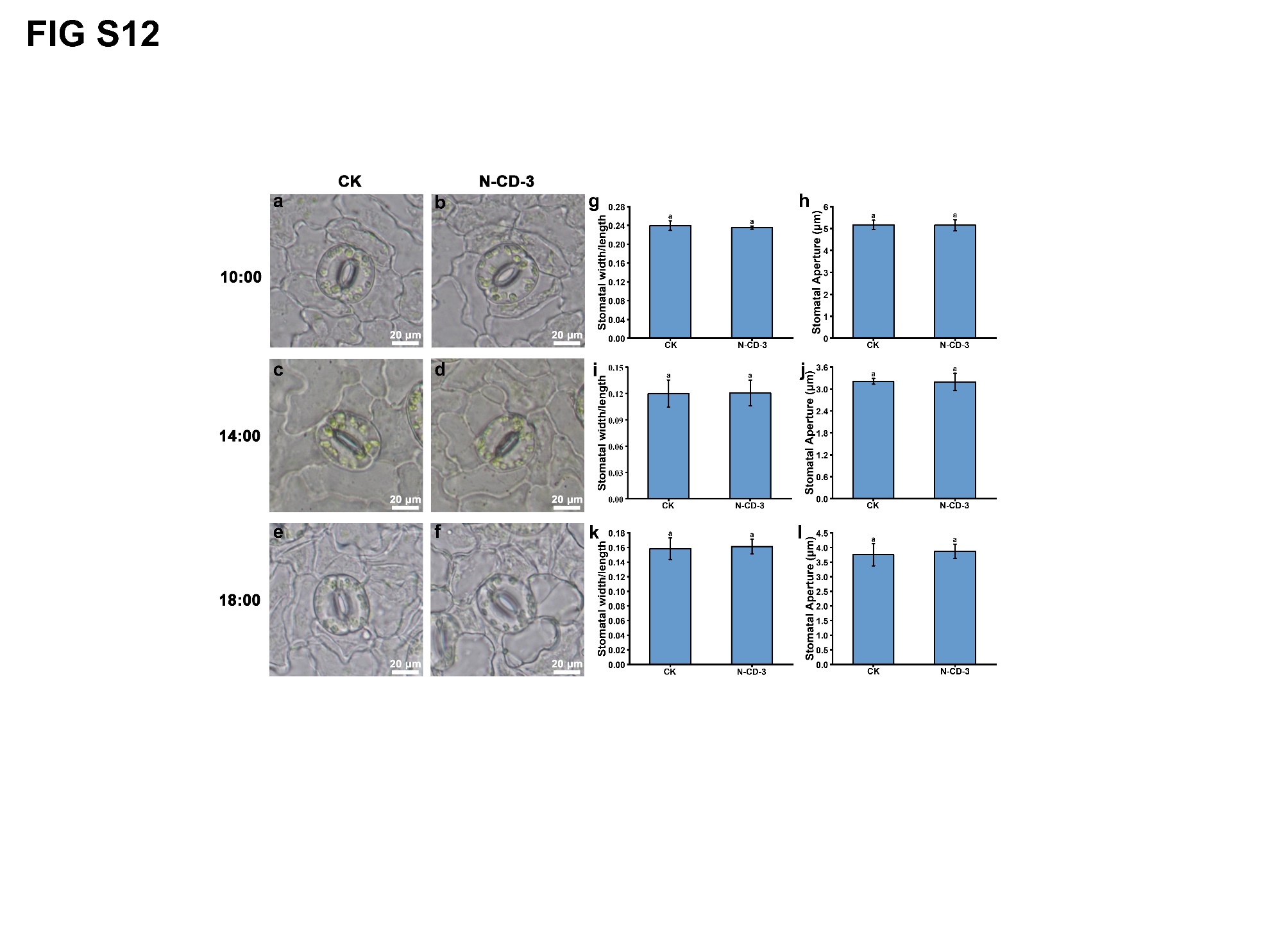


**Figure S11.** Stomatal aperture of *Malus hupehensis* seedlings with or without N-CD-3 treatment after a period. (a-f) The photomicrograph of stomata of *Malus hupehensis* seedlings at 10:00, 14:00 and 18:00 with or without N-CD-3 treatment. (g,i,k) Calculated stomatal width/length ratio of seedlings in different periods with or without N-CD-3 treatment. (h,j,l) Stomatal aperture is measured under the microscope of seedlings in different periods with or without N-CD-3 treatment. One-way analysis of variance (ANOVA) was used to analyze all data and error bars represent the SD from more than three biological replicates with three parallel experiments. Different lowercase letters represent significant differences (*P<0.05*, Tukey test).


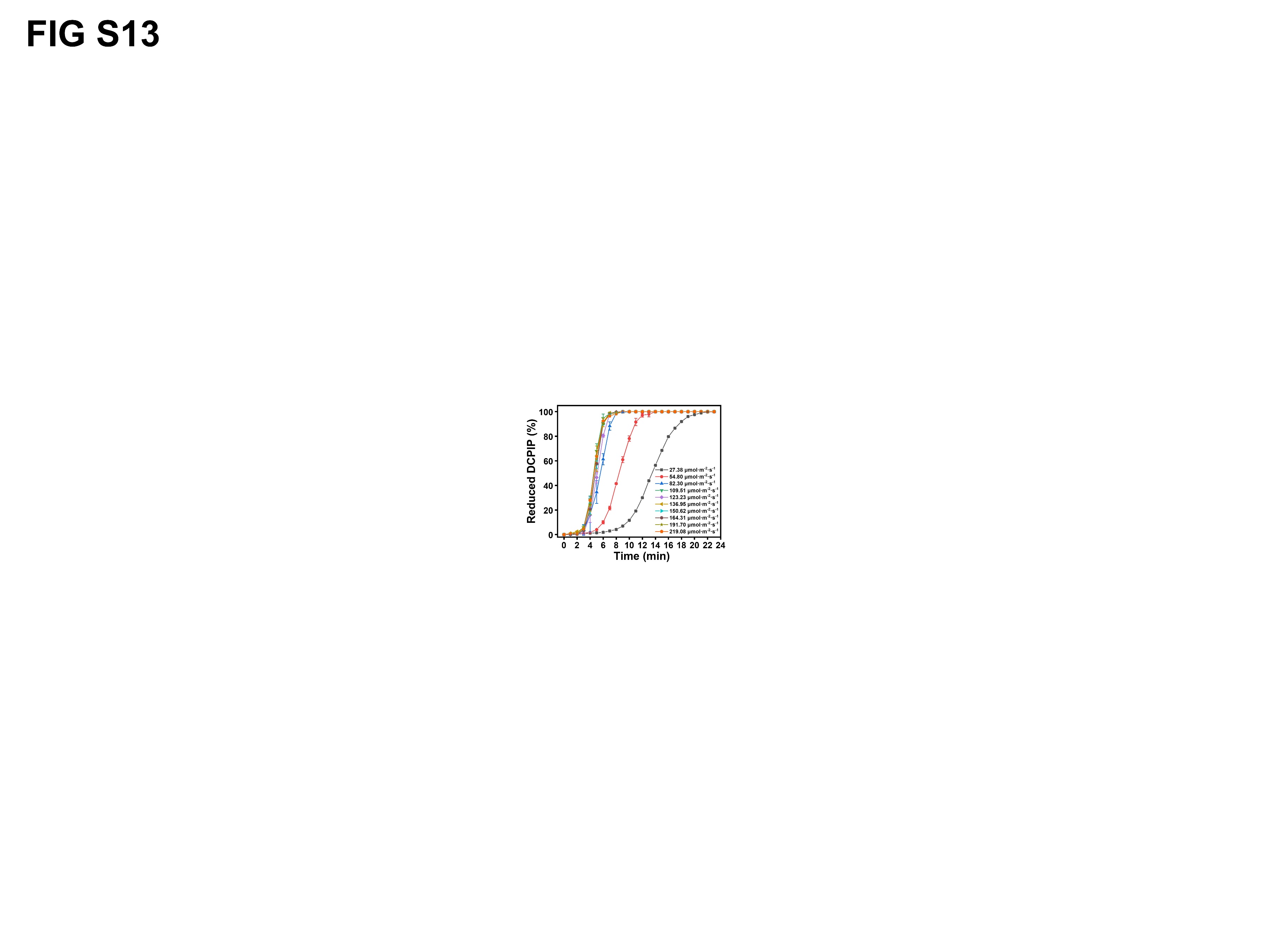


**Figure S12.** Hill reaction activity of isolated chloroplasts at different light intensities.


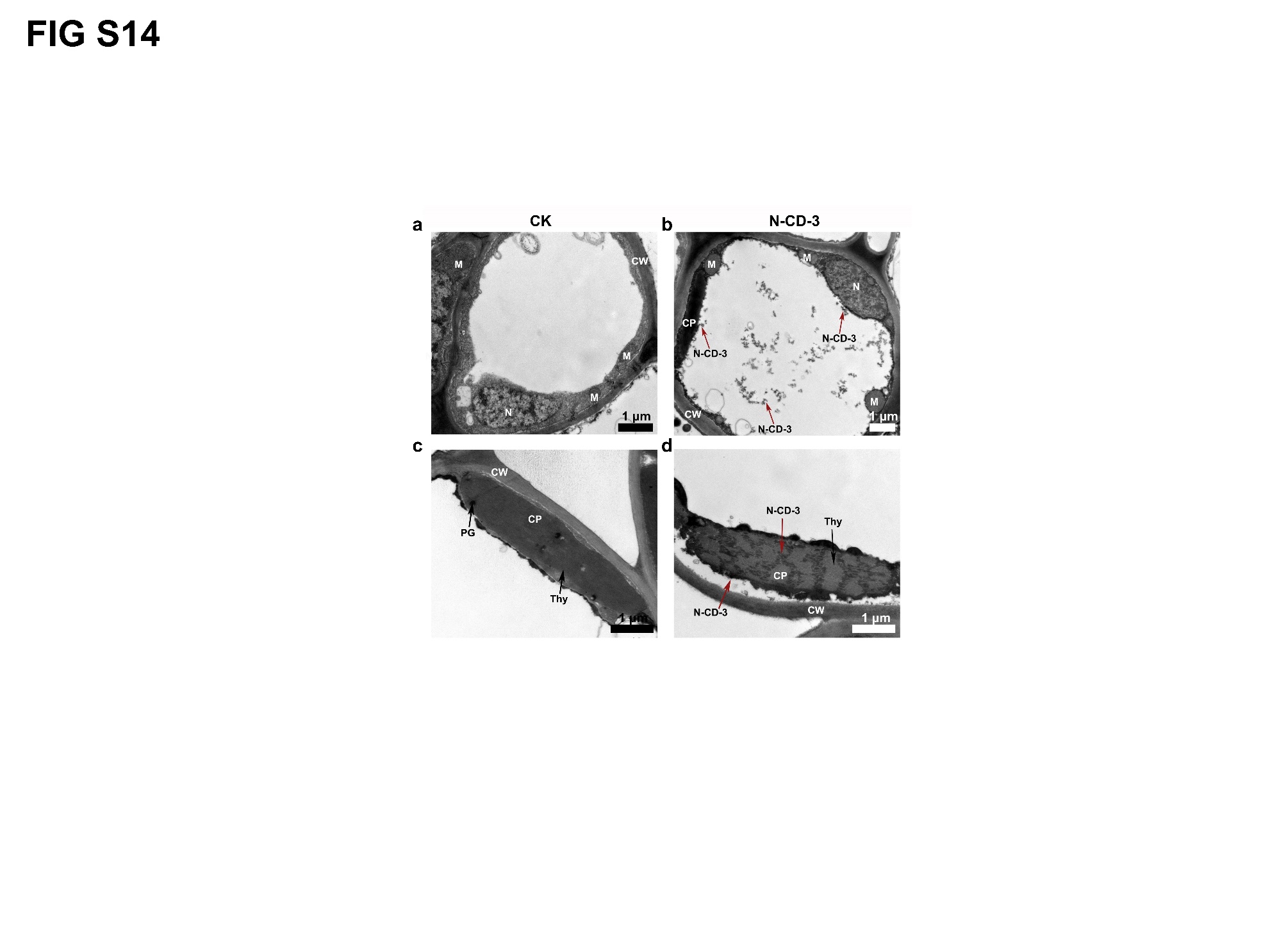


**Figure S13.** Bio-TEM images in *Malus hupehensis* seedling leaves (a,b) and chloroplast (c,d) with (b,d) /without (a,c) the treatment of 300 mg·L^-1^ N-CD-3. N-CD-3-enriched regions, where numerous black particles were found, presented a darker color (b,d) compared to the control group (a,c). Abbreviations: Cell wall (CW), Cell nucleus (N), Mitochondrion (M), Chloroplast (CP), Plastoglobuli (PG), thylakoid (Thy).


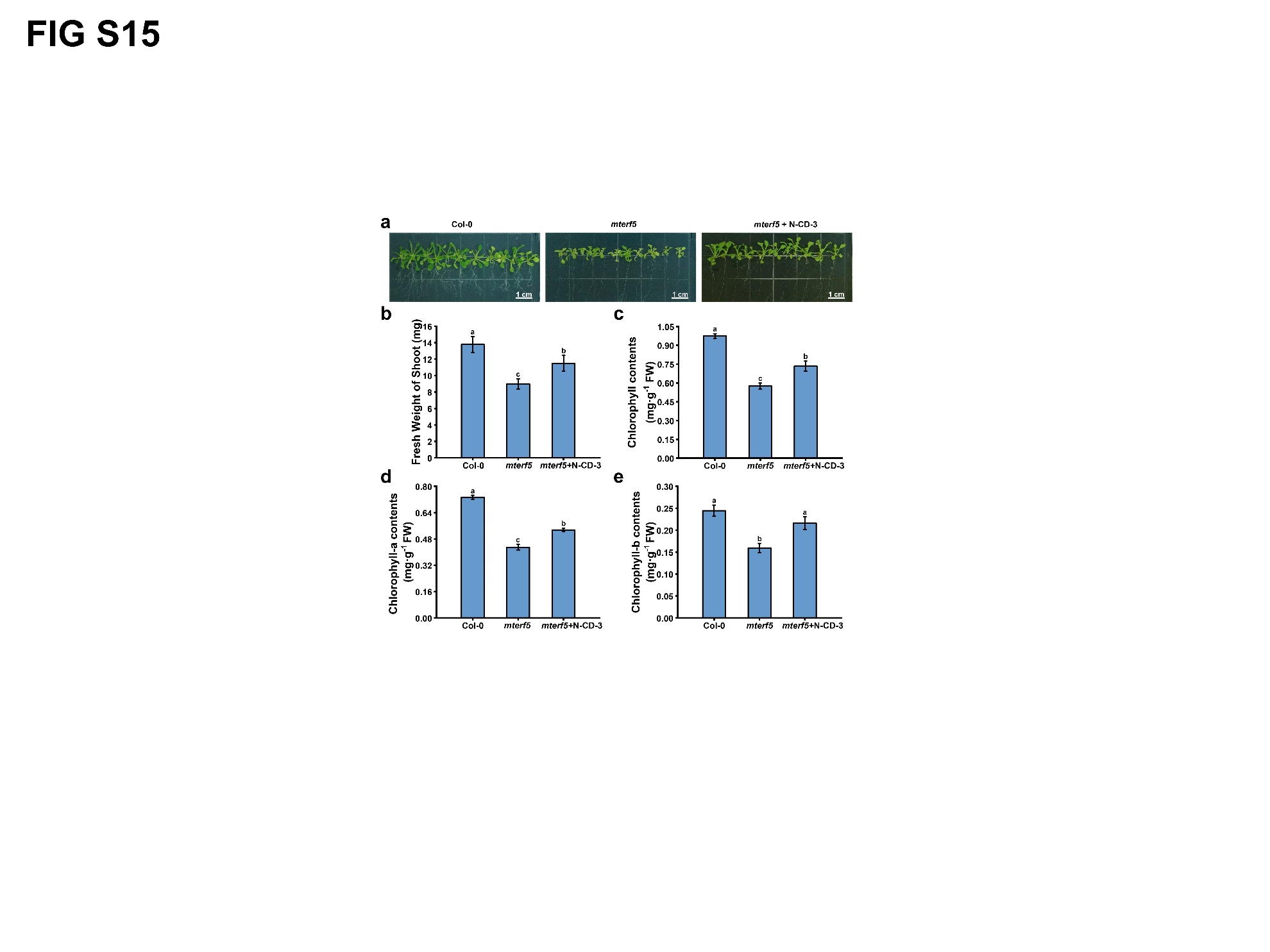


**Figure S14.** N-CD-3 complements the PSII system. (a) Effects of N-CD-3 (300 mg·L^-1^) on the Col-0, *mterf5* mutants in 1/2 MS medium (1% sucrose) under light illumination (20.43 μmol·m^-2^·s^-1^) containing UV-A (λ_max_ 365 nm, 0.83 μmol·m^-2^·s^-1^). Pictures captured after 14 d of cultivation. (b-e) The fresh weight (b), chlorophyll content (c), chlorophyll a content (d), chlorophyll b content (e) with/without the N-CDs treatment of seedlings. One-way analysis of variance (ANOVA) was used to analyze all data and error bars represent the SD from three biological replicates with three parallel experiments. Different lowercase letters represent significant differences (*P<0.05*, LSD test).


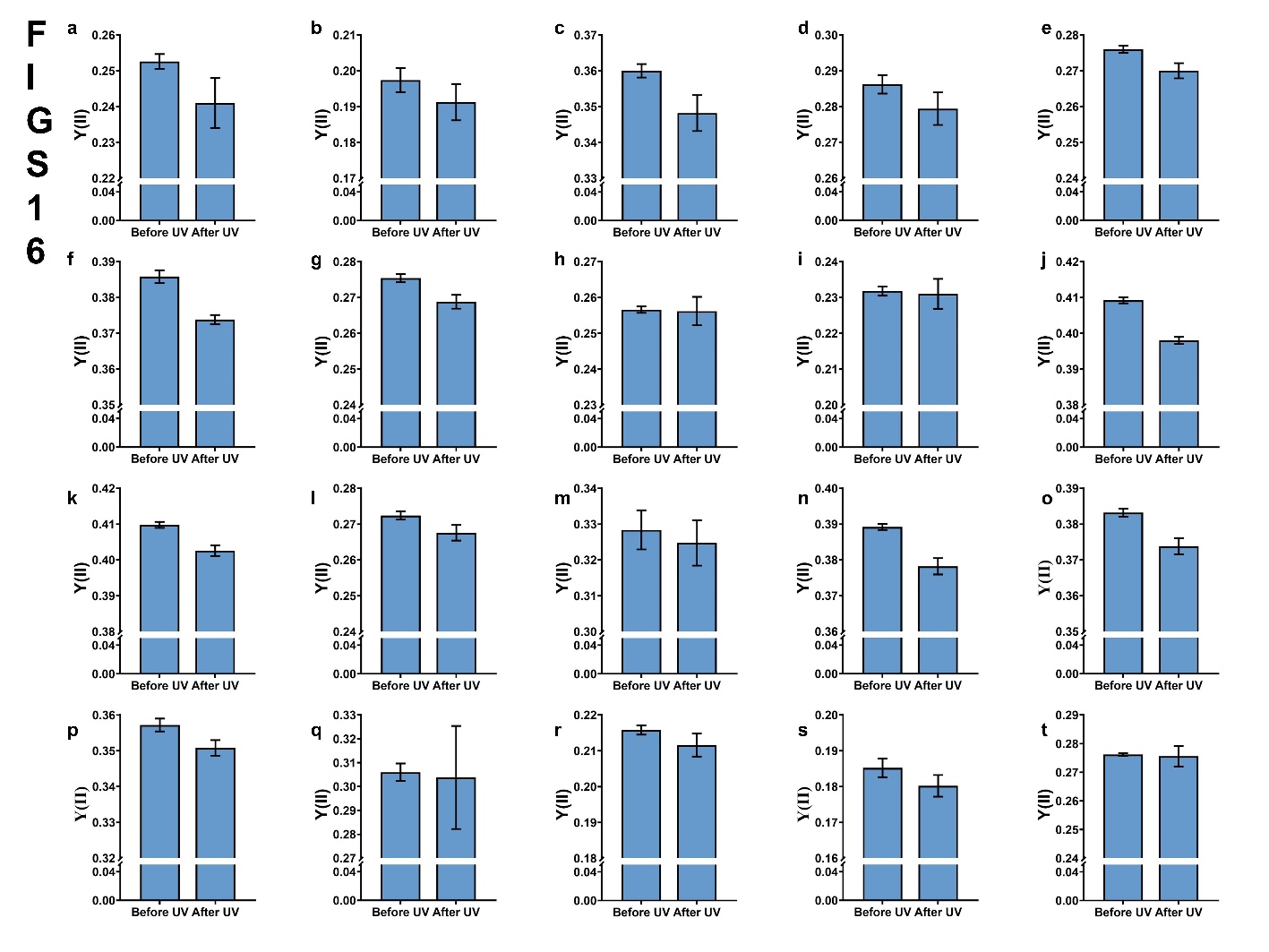


**Figure S15.** The Y(II) values of leaves in the control group under the activation light illumination before and after the addition of UV-A light (λ_max_ 365 nm, 2.48 μmol·m^-2^·s^-1^) in the measurement process. Data are mean ± SD of the three record points after numerical stabilization.


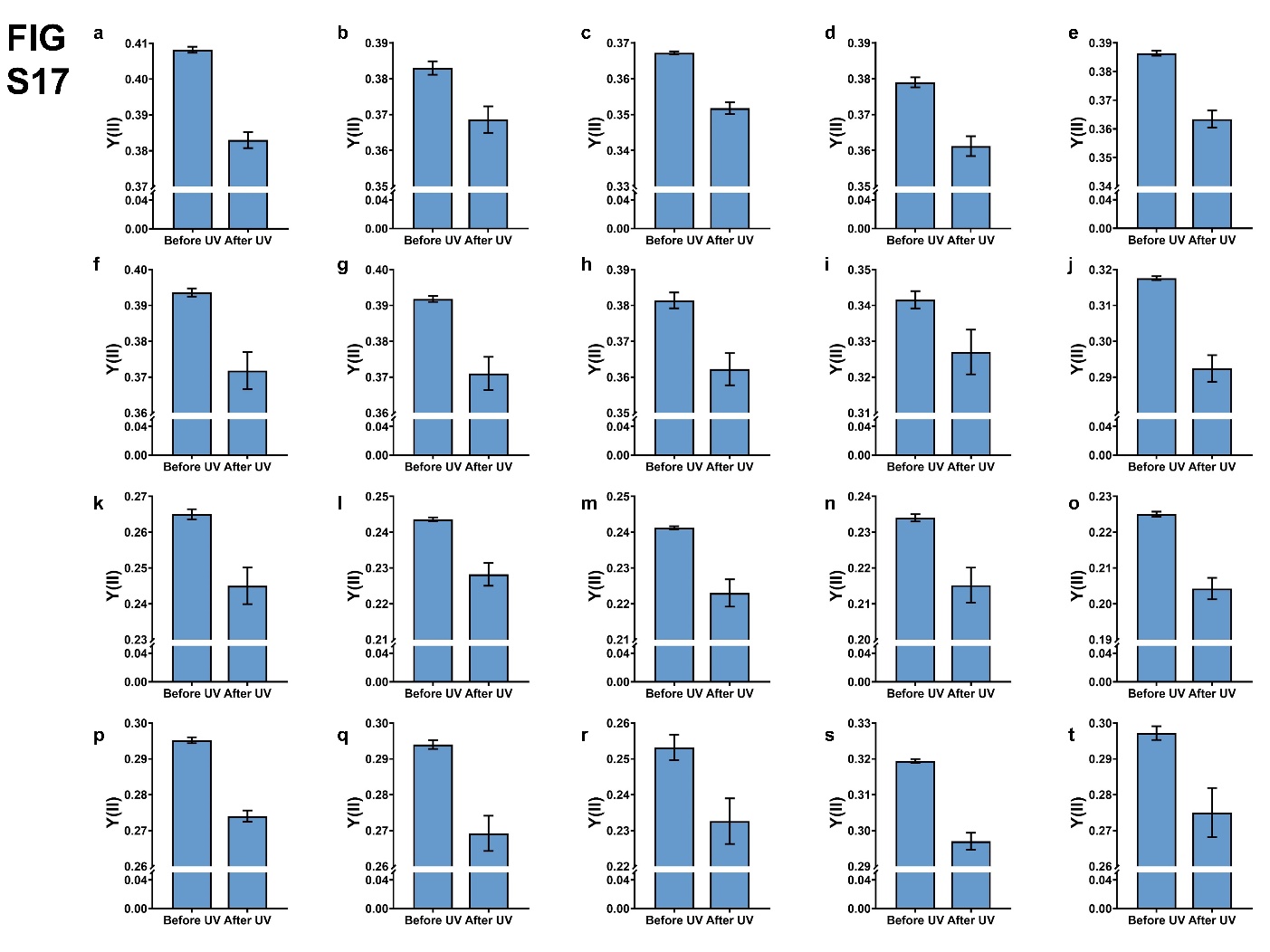


**Figure S16.** The Y(II) values of N-CD-3-treated leaves under the activation light illumination before and after the addition of UV-A light (λ_max_ 365 nm, 2.48 μmol·m^-2^·s^-1^) in the measurement process. Data are mean ± SD of the three record points after numerical stabilization.


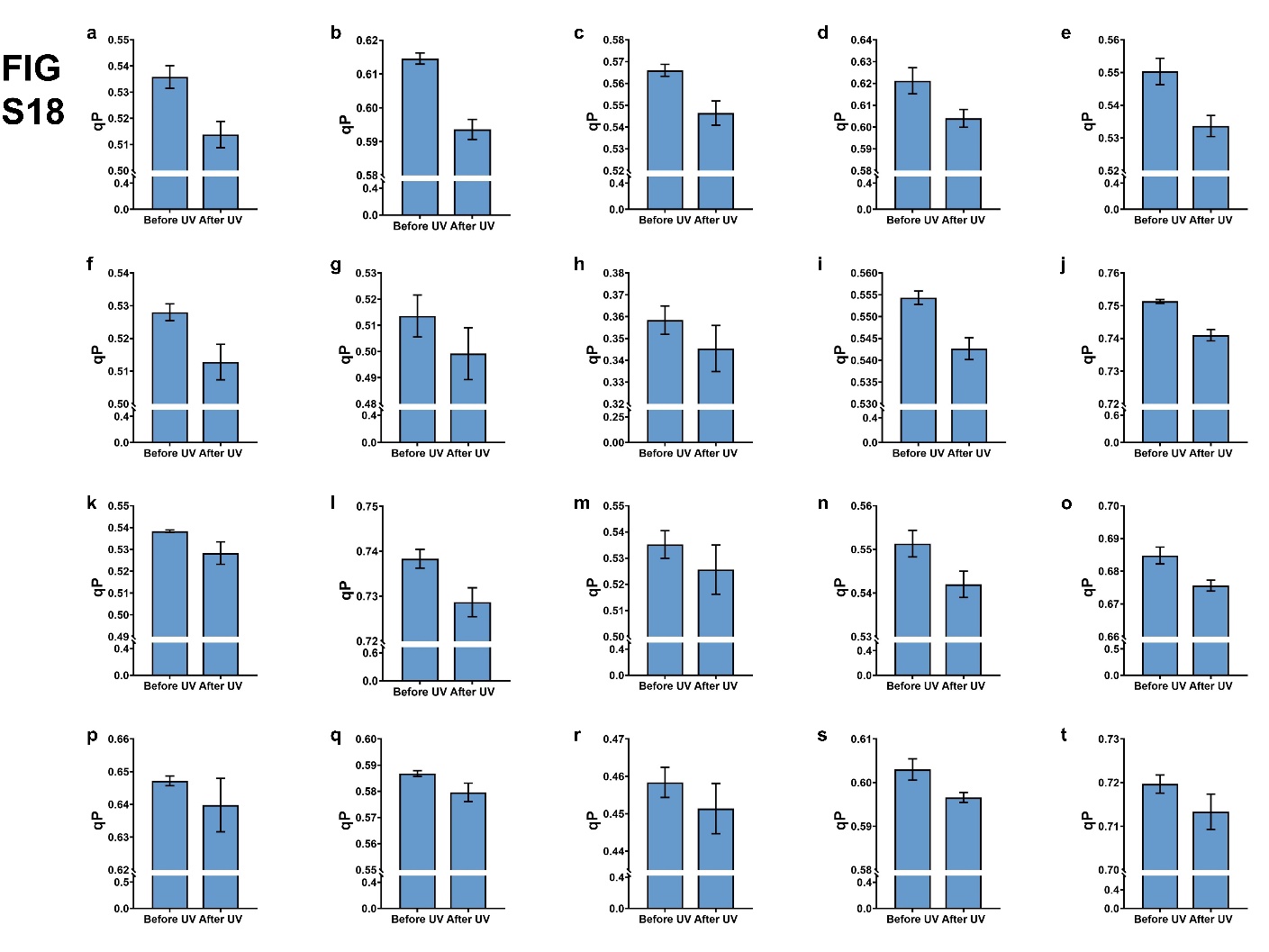


**Figure S17.** The qP values of leaves in the control group under the activation light illumination before and after the addition of UV-A light (λ_max_ 365 nm, 2.48 μmol**·**m^-2^**·**s^-1^) in the measurement process. Data are mean ± SD of the three record points after numerical stabilization.


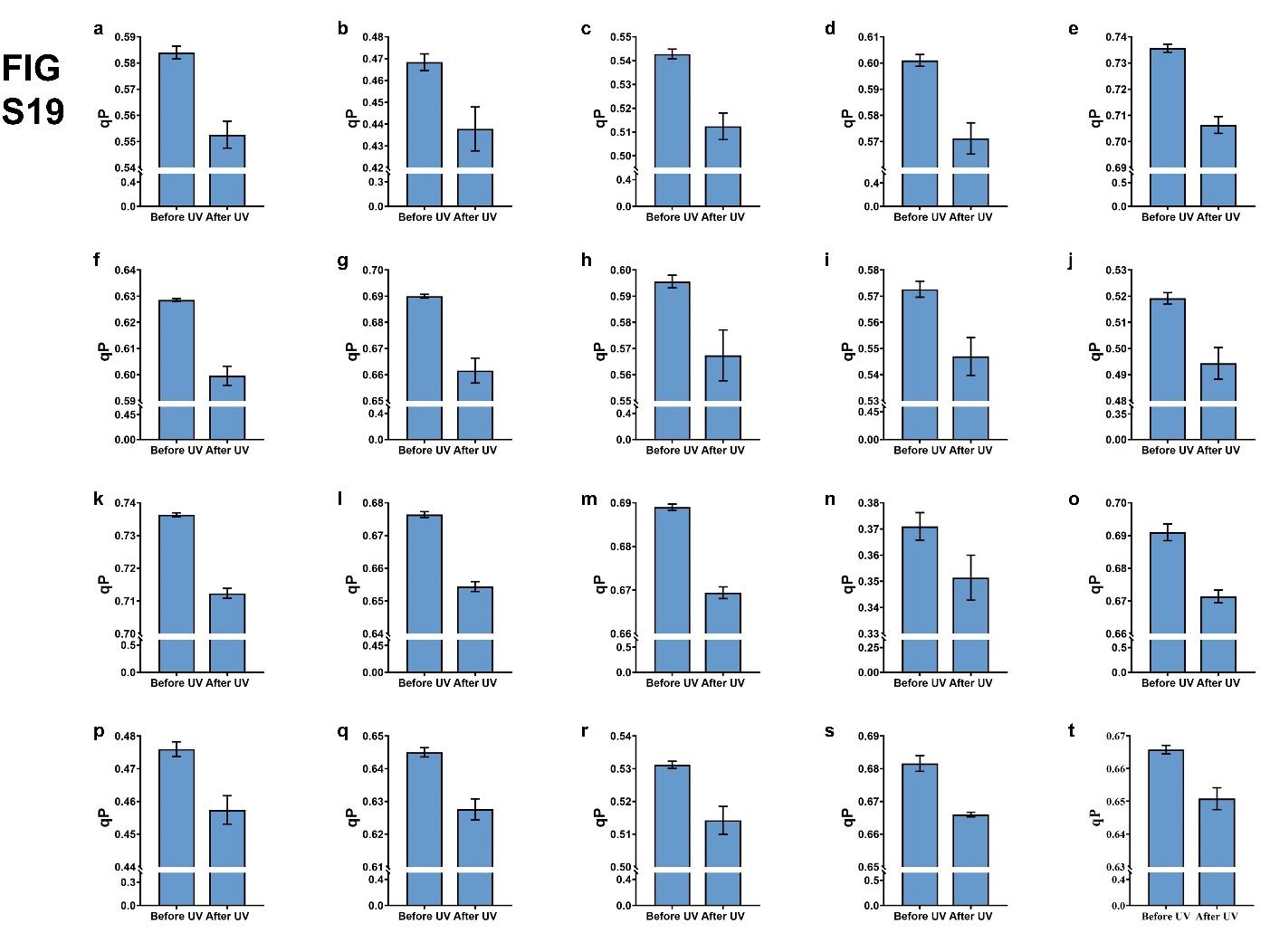


**Figure S18.** The qP values of N-CD-3-treated leaves under the activation light illumination before and after the addition of UV-A light (λ_max_ 365 nm, 2.48 μmol·m^-2^·s^-1^) in the measurement process. Data are mean ± SD of the three record points after numerical stabilization.

**
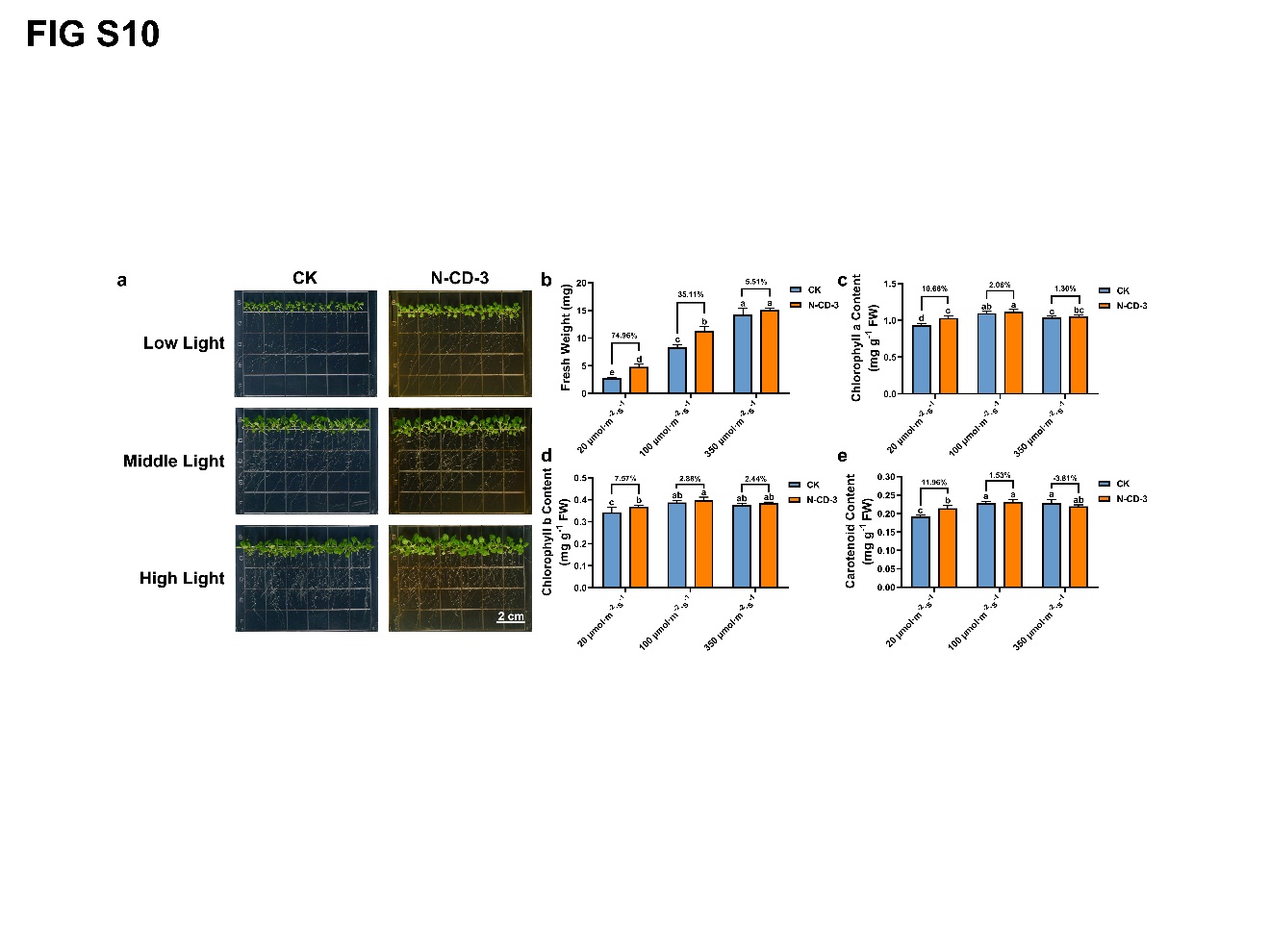
**

**Figure S19.** Effects of N-CD-3 on the *Arabidopsis thaliana* seedlings. (a) Effects of N-CD-3 (300 mg·L^-1^) on the seedlings under different light intensities (low light: 20 μmol·m^-2^·s^-1^, middle light: 100 μmol·m^-2^·s^-1^, high light: 350 μmol·m^-2^·s^-1^) containing UV-A (λ_max_ 365 nm, 0.83 μmol·m^-2^·s^-1^). Pictures captured after 10 d of cultivation. (b-e) The fresh weight (b), chlorophyll a content (c), chlorophyll b content (d), carotenoid content (e) with/without the N-CD-3 treatment of seedlings. One-way analysis of variance (ANOVA) was used to analyze all data and error bars represent the SD from more than three biological replicates with three parallel experiments. Different lowercase letters represent significant differences (*P*<0.05, Tukey test).

**
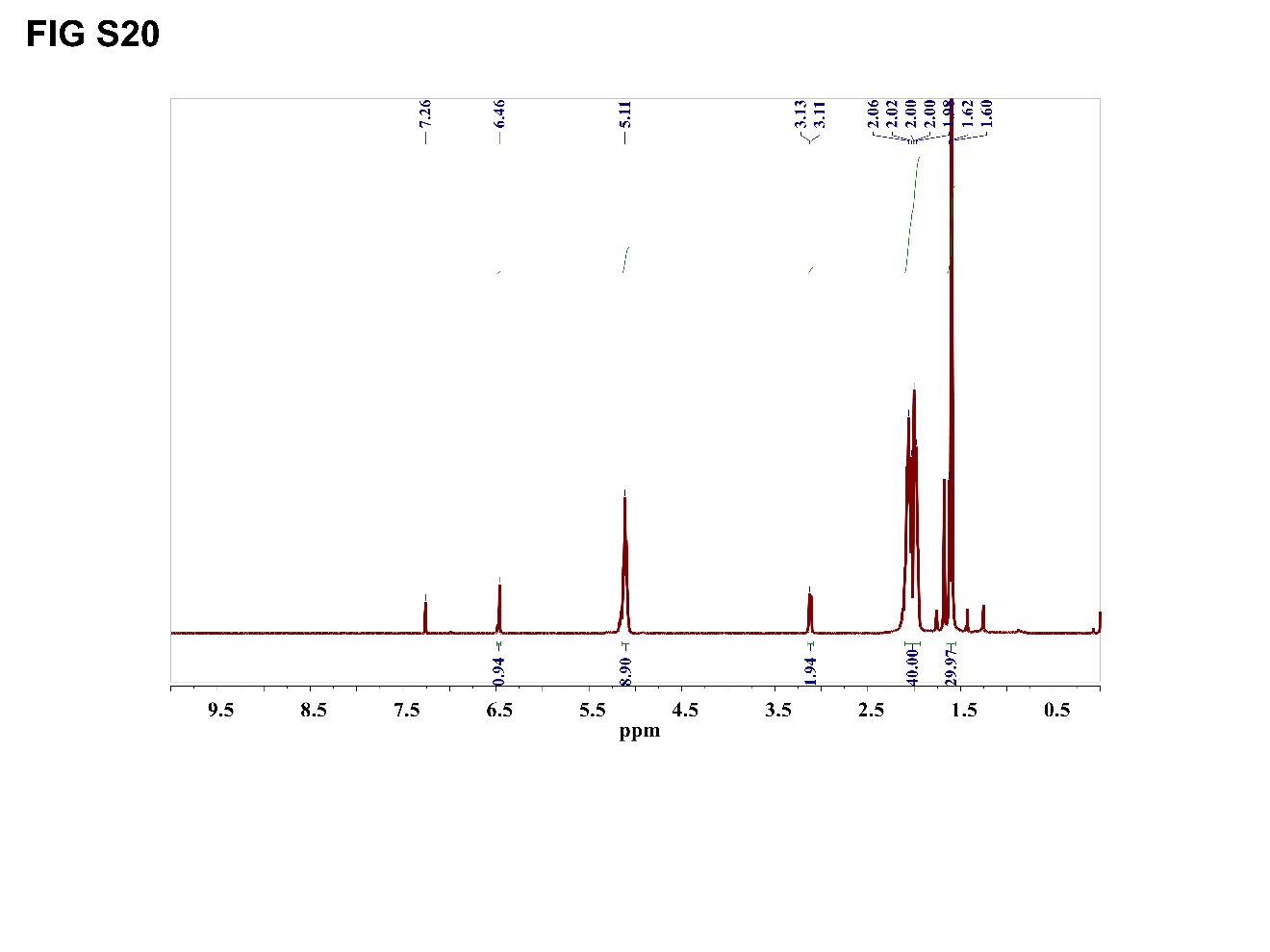
**

**Figure S20.** ^1^H NMR of PQ-9.


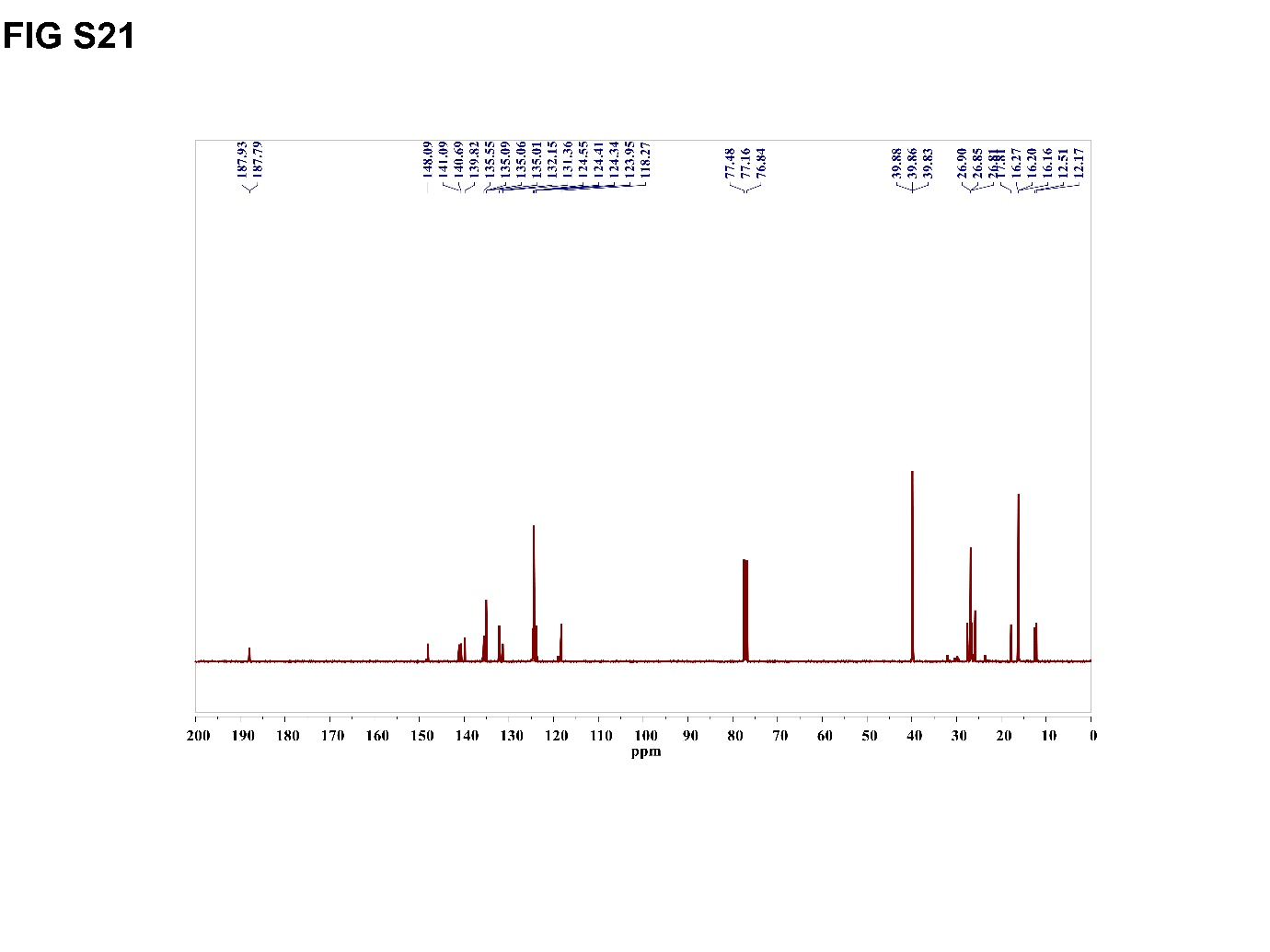


**Figure S21.** ^13^C NMR of PQ-9.


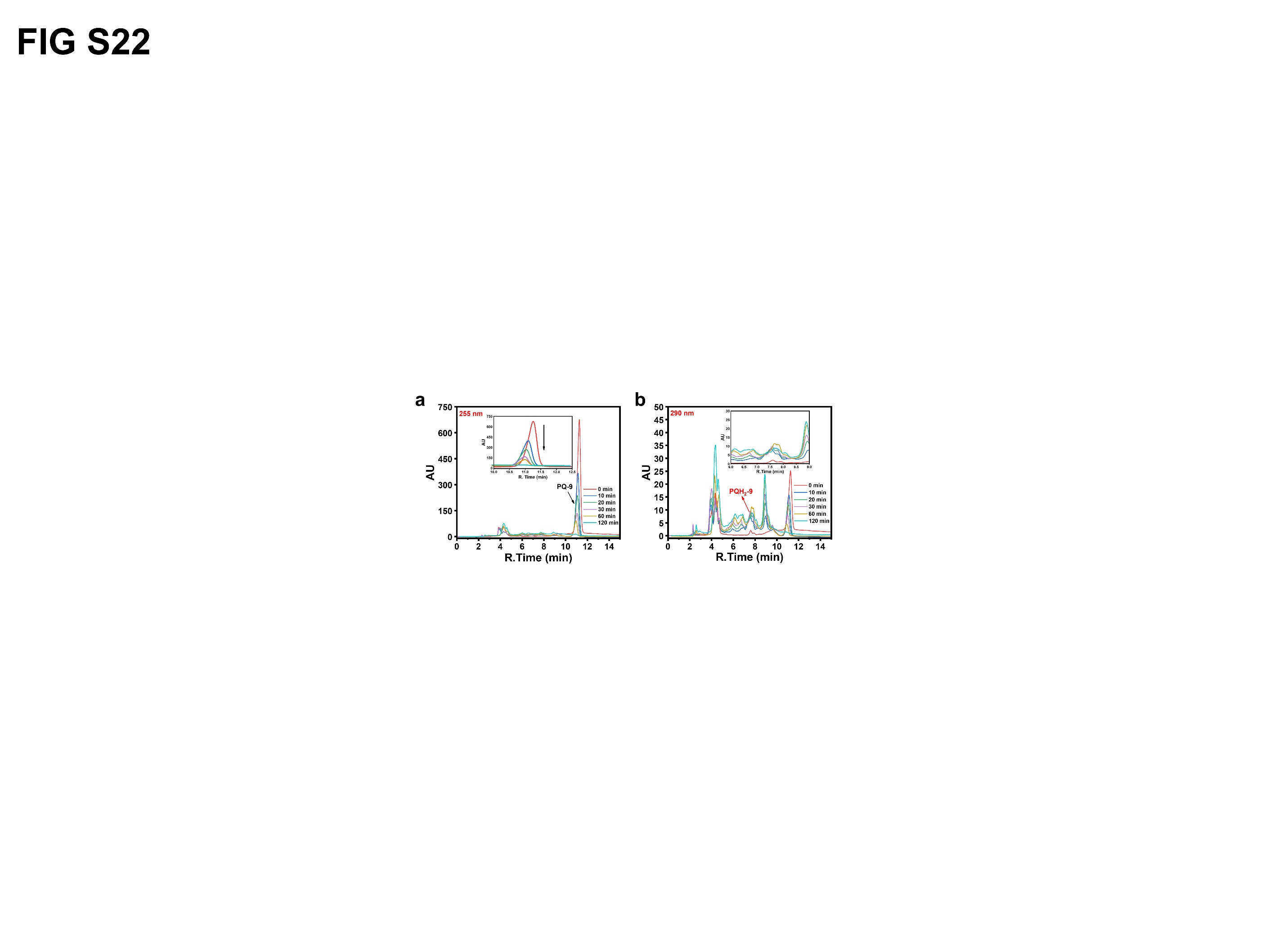


**Figure S22.** The absorbance changes of PQ-9 (255 nm, 11.42 min) (a) and PQH_2_-9 (290 nm, 7.57 min) (b) under UV-A light in THF/H_2_O (V: V = 2:1) solution (PQ-9: 0.2 g·L^-1^) without N-CD-3.


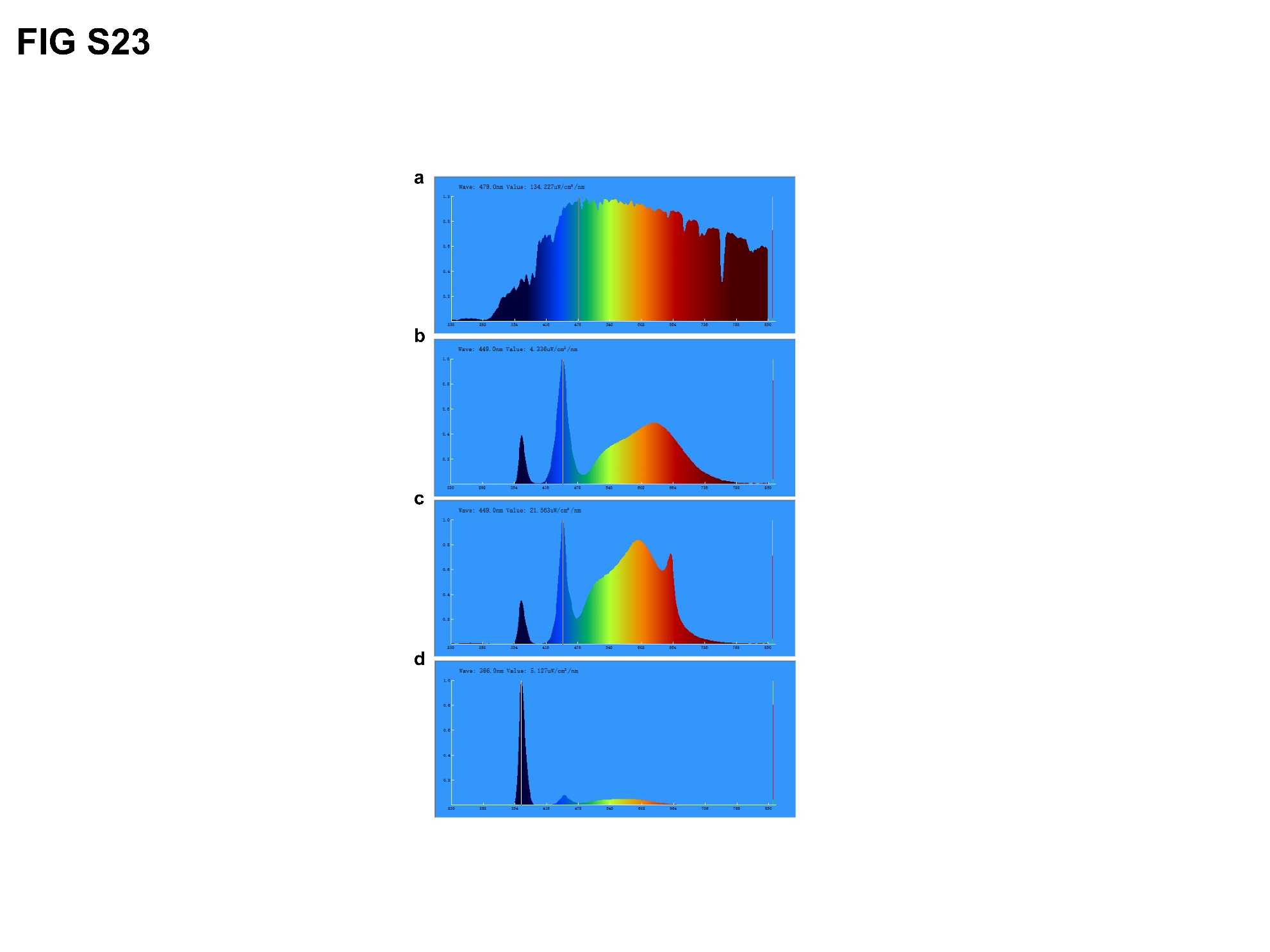


**Figure S23.** Light spectra used in this work. (a) Sunny sunlight. (b) Seedlings growth light spectrum containing UV-A light (λ_max_ 365 nm). (c) Light spectrum used in the measurement of Hill reaction*.* (d) Light spectrum used in the measurement of Y(II) and qP value changes and PQ-9 reduction.
